# Calculation and Interpretation of Substrate Assimilation Rates in Microbial Cells Based on Isotopic Composition Data Obtained by nanoSIMS

**DOI:** 10.3389/fmicb.2021.621634

**Published:** 2021-11-30

**Authors:** Lubos Polerecky, Meri Eichner, Takako Masuda, Tomáš Zavřel, Sophie Rabouille, Douglas A. Campbell, Kimberly Halsey

**Affiliations:** ^1^Department of Earth Sciences, Utrecht University, Utrecht, Netherlands; ^2^Institute of Microbiology, Czech Academy of Sciences, Centre Algatech, Třeboň, Czechia; ^3^Global Change Research Institute, Czech Academy of Sciences, Brno, Czechia; ^4^Sorbonne Université, CNRS, Laboratoire d’Océanographie de Villefranche, LOV, Villefranche-sur-mer, France; ^5^Sorbonne Université, CNRS, Laboratoire d’Océanographie Microbienne, LOMIC, Banyuls-sur-mer, France; ^6^Department of Biology, Mount Allison University, Sackville, NB, Canada; ^7^Department of Microbiology, Oregon State University, Corvallis, OR, United States

**Keywords:** nanoSIMS, stable isotope probing, assimilation rates, storage inclusions, cell growth model

## Abstract

Stable isotope probing (SIP) combined with nano-scale secondary ion mass spectrometry (nanoSIMS) is a powerful approach to quantify assimilation rates of elements such as C and N into individual microbial cells. Here, we use mathematical modeling to investigate how the derived rate estimates depend on the model used to describe substrate assimilation by a cell during a SIP incubation. We show that the most commonly used model, which is based on the simplifying assumptions of linearly increasing biomass of individual cells over time and no cell division, can yield underestimated assimilation rates when compared to rates derived from a model that accounts for cell division. This difference occurs because the isotopic labeling of a dividing cell increases more rapidly over time compared to a non-dividing cell and becomes more pronounced as the labeling increases above a threshold value that depends on the cell cycle stage of the measured cell. Based on the modeling results, we present formulae for estimating assimilation rates in cells and discuss their underlying assumptions, conditions of applicability, and implications for the interpretation of intercellular variability in assimilation rates derived from nanoSIMS data, including the impacts of storage inclusion metabolism. We offer the formulae as a Matlab script to facilitate rapid data evaluation by nanoSIMS users.

## Introduction

Stable isotope probing (SIP) is an experimental approach used to trace the fates of substrates within a microbial community. In this approach, samples are incubated with a target substrate that is enriched in a stable isotope, such as ^13^C, and then cells or cellular components are analyzed for enrichment in the target isotope (Boschker and Middelburg, 2002; Dumont and Murrell, 2005; Neufeld et al., 2007; Hungate et al., 2015). SIP can be used to identify microbes in environmental samples that are actively using a particular growth substrate by selectively recovering and analyzing isotopic signatures in biomarkers such as DNA, rRNA, lipids, fatty acids or proteins (Boschker and Middelburg, 2002; Neufeld et al., 2007; Jehmlich et al., 2016). SIP can also be used to quantify rates of substrate assimilation and compartmentalization by specific community members with the goal of estimating fluxes at the ecosystem scale (Dumont and Murrell, 2005; Hungate et al., 2015). In the latter application, use of nano-scale secondary ion mass spectrometry (nanoSIMS; see Hoppe et al., 2013 or Nuñez et al., 2017 for a review of the method) to measure substrate assimilation into individual cells has become popular in the last decade (Musat et al., 2012; Pett-Ridge and Weber, 2012; Mayali, 2020; Ploug, 2021). Coupling SIP with nanoSIMS enables measurement of activities of microbes from the environment, including those that cannot yet be cultured (Krupke et al., 2015; Harding et al., 2018; Dekas et al., 2019; Geerlings et al., 2020; Mills et al., 2020), are rare (Musat et al., 2008; Zimmermann et al., 2015), or are tightly associated with their geophysical and/or biological environments (Foster et al., 2011, 2013; Milucka et al., 2012; Bonnet et al., 2016; Berthelot et al., 2019; Loussert-Fonta et al., 2020), thus offering opportunities to advance understanding of how single-celled life works, adapts, and impacts geochemical element cycling.

A typical output of a nanoSIMS measurement is the isotopic composition of a cell, determined as the cell-specific isotope ratio, *R*, or atom fraction, *x* [e.g., for carbon isotopes, *R* = ^13^*C*/^12^*C* and *x* = ^13^*C*/(^12^*C*+^13^*C*)]. One way of using this data is to convert *R* or *x* into quantities that can directly be interpreted in terms of the cell’s mass balance, including *Fx*_*net*_, *K*_*A*_ or *X*_*net*_ (Popa et al., 2007; Finzi-Hart et al., 2009; Stryhanyuk et al., 2018; Dekas et al., 2019). Specifically, the parameter *Fx*_*net*_, introduced by Popa et al. (2007) and equal to the parameter *K*_*A*_ introduced by Stryhanyuk et al. (2018), reflects the net amount of element (e.g., carbon) assimilated by the cell during the SIP experiment (*E*_*a*_) relative to the initial content of the element in the cell prior to the SIP experiment (*E*_*i*_), i.e., *Fx*_*net*_ = *K*_*A*_ = *E*_*a*_/*E*_*i*_. Similarly, the parameter *X*_*net*_, used for instance by Dekas et al. (2019), reflects the net amount of element assimilated by the cell relative to the final element content in the cell (*E*_*f*_), i.e., *X*_*net*_ = *E*_*a*_/*E*_*f*_ (Table 1).

**TABLE 1:** List of symbols used in this study.

Symbol	Unit	Description
*C*, *C*_*i*_	mol C cell**^–^**^1^	Carbon content of a cell; ‘i’ refers to the initiation of the SIP incubation, i.e., time-point *t* = 0.
*C*_*max*_	mol C cell**^–^**^1^	Carbon content of a cell just before binary cell division; *C*_*max*_/2 corresponds to the carbon content just after binary cell division.
*s*	-	Cell cycle stage, calculated from the C content as *s* = *C*/(*C*_*max*_/2) – 1.
⟨*C*⟩	mol C cell**^–^**^1^	Carbon content of a cell *averaged* over the cell cycle. It is equal to the average in a population of cells with perfectly unsynchronized cell cycles. It is related to *C*_*max*_ according to ⟨*C*⟩ = *C*_*max*_/(2⋅*ln*(2)) and ⟨*C*⟩ = *C*_*max*_⋅*ln*(2) for cells assimilating C with zero-order and first-order kinetics, respectively (Koch, 1966).
*x*, *x*_*i*_	-	^13^C atom fraction of a cell, defined as *x* = ^13^*C*/(^12^*C+*^13^*C*); ‘i’ refers to *t* = 0.
$x_{S}^{E}$	-	Source-normalized excess ^13^C atom fraction of a cell (Eq. 10).
*r*	mol C cell**^–^**^1^ h**^–^**^1^	Cell-specific rate of carbon assimilation by a cell.
⟨*r*⟩	mol C cell**^–^**^1^ h**^–^**^1^	Average cell-specific C assimilation rate in a population.
*k*	h**^–^**^1^	Carbon-specific rate of carbon assimilation by a cell.
*t*	h	Doubling time; time needed for a cell to double its carbon content. Calculated as τ = *C*_*max*_/(2⋅*r*) and τ=(*ln*2)/*k* for a cell assimilating C with zero-order and first-order kinetics, respectively.
ρ	mol C μm**^–^**^3^	Carbon density of a cell.
*x*_*S,tar*_	-	^13^C atom fraction of the target source provided externally during the SIP incubation.
*x*_*S,alt*_	-	^13^C atom fraction of an alternative carbon source; can be external (e.g., present in the environment of the cell) or internal (present in the form of intra-cellular C storage inclusions).
*x*_*S,eff*_	-	Effective ^13^C atom fraction of the carbon source (Eq. 6).
*f*_*tar*_	-	Fraction of C assimilated by the cell from the target source; the remaining fraction of C is assimilated from the alternative C source (*f*_*alt*_ = 1 – *f*_*tar*_).
θ	-	Probability density function (PDF) describing the distribution of *C* among cells in a population. Given by Eq. 16 and 18 for a population with perfectly unsynchronized and partially synchronized cell cycles, respectively. Examples shown in Figure 4B, Supplementary Figures 3 and 4).
ζ	-	Probability density function (PDF) describing the distribution of $x_{S}^{E}$ in a population of cells assimilating C with zero-order kinetics. Examples shown in Figure 4C.
γ	-	Degree of cell cycle synchronicity characterizing the distribution of C content among cells in a population with partially synchronized cell cycles. Examples shown in Supplementary Figures 4A,B.
*X*_*net*_	-	Net amount of element assimilated by the cell (*E*_*a*_) relative to the *final* element content in the cell (*E*_*f*_). Calculated according to *X*_*net*_ = *E*_*a*_/*E*_*f*_. Used, for instance, by Dekas et al. (2019). For a cell that did not divide during the SIP incubation, *X*_*net*_ = $x_{S}^{E}$ (Supplementary Material, Section “Relationships Between $x_{S}^{E}$, *X*_*net*_, *Fx*_*net*_ and *K*_*A*_.”).
*Fx*_*net*_, *K*_*A*_	-	Net amount of element assimilated by the cell (*E*_*a*_) relative to the *initial* element content in the cell (*E*_*i*_). Calculated according to *Fx*_*net*_ = *K_*A*_* = *E*_*a*_/*E*_*i*_. Parameter *Fx*_*net*_ was introduced by Popa et al. (2007), while parameter *K*_*A*_ was introduced by Stryhanyuk et al. (2018). For a cell that did not divide during the SIP incubation, *Fx*_*net*_ = *K_*A*_* = $x_{S}^{E}/\left(1-x_{S}^{E}\right)$ (Supplementary Material, Section “Relationships Between $x_{S}^{E}$, *X*_*net*_, *Fx*_*net*_ and *K*_*A*_.”).

The quantities described above do not account for incubation duration so can only be compared across SIP experiments that are incubated for the same duration. Calculating the rate of substrate assimilation obviates this requirement and has become a common output from nanoSIMS analyses of microbial cells (Ploug, 2021, and references therein). The two types of substrate assimilation rates used are the substrate-specific and cell-specific assimilation rates. While the substrate-specific rate [e.g., the rate of carbon assimilation normalized to the carbon content of the cell; in mol C (mol C)^–1^ h^–1^] provides useful information on cellular turnover of the substrate and can be converted to a cell’s doubling time (e.g., Musat et al., 2008; Martínez-Pérez et al., 2016; Eichner et al., 2017), the cell-specific rate (e.g., the carbon assimilation rate per cell; in mol C cell^–1^ h^–1^) can be used to estimate the impact of a cell’s population on element fluxes over larger spatial and temporal scales (e.g., Foster et al., 2011; Krupke et al., 2015; Bonnet et al., 2016; Klawonn et al., 2016; Arandia-Gorostidi et al., 2017; Mills et al., 2020).

Calculating substrate assimilation rates by individual cells from nanoSIMS data requires consideration of cell division and the mathematical description of cell growth. Substrate assimilation during a SIP incubation, as indicated by isotopic enrichment of cell biomass at the end of the incubation (i.e., a positive value of the cell-specific *Fx*_*net*_ or *X*_*net*_), implies that there is a non-zero probability that the cell divided during the SIP incubation. A simple mass balance calculation reveals that, under balanced growth, this probability reaches 1 when the cell-specific quantities *Fx*_*net*_ and *X*_*net*_ exceed 1 and 0.5, respectively. Some recent studies have considered cell division occurring during SIP incubations by applying a correction factor (i.e., 2) in growth rate calculations (Schoffelen et al., 2018, 2019; Olofsson et al., 2019a,b). The mathematical basis for such correction factors has not been, however, explicitly formulated, and the impact of cell division on calculated rates of substrate assimilation in cells has not been investigated in a systematic way.

Calculated substrate assimilation rates will vary depending on the model used to describe substrate assimilation over the cell cycle (i.e., cell growth) and thus over the incubation time. A linear model assumes that substrate assimilation proceeds at a constant cell-specific rate (i.e., zero-order kinetics) leading to a linear increase in the biomass of individual cells over time. In contrast, an exponential model assumes that substrate assimilation proceeds at a rate that is linearly proportional to the instantaneous cell biomass (i.e., first-order kinetics) leading to an exponential increase in the biomass of individual cells over time (Collins and Richmond, 1962; Koch and Schaechter, 1962; Koch, 1966). While the linear model of substrate assimilation is most commonly used to calculate assimilation rates in cells from nanoSIMS data (e.g., Foster et al., 2013; Klawonn et al., 2016; Stryhanyuk et al., 2018; Mills et al., 2020), the exponential model is used rather rarely (e.g., Martínez-Pérez et al., 2016; Berthelot et al., 2019; Geerlings et al., 2020, 2021; Polerecky et al., 2021). The impact of the substrate assimilation model (linear vs. exponential) on the calculated assimilation rate has previously not been studied in detail.

Assimilation rates determined at single-cell resolution often reveal intercellular heterogeneity in populations. Previous nanoSIMS studies typically attributed such heterogeneity to factors that affect the *assimilation* of an (isotopically labeled) element during the incubation (causing variation in *E*_*a*_), i.e., differences in the intrinsic metabolic activity of a cell, which may vary with cell cycle stage or cell age, or stochastic gene expression (e.g., reviewed by Ackermann, 2015). Other sources of heterogeneity have received less attention, such as assimilation of unlabeled sources of the target element, which will affect the degree of isotopic labeling of the assimilated material, or differences in the cell’s initial elemental content (variation in *E*_*i*_). For instance, selective assimilation of C and N into storage inclusions or mobilization of C or N from existing cell material during a SIP experiment can critically affect both the (apparent) amount of an element assimilated during an incubation (*E*_*a*_) and the initial amount of an element present in the cell (*E*_*i*_) (Polerecky et al., 2021). Thus, metabolism of storage compounds can partly explain variability in the enrichment of cellular biomass (e.g., *Fx*_*net*_ or *X*_*net*_).

In this work we use mathematical modeling to identify key processes and parameters that play a role when calculating and interpreting cellular substrate assimilation rates from the isotopic composition information determined by SIP-nanoSIMS. First, we investigate how the isotopic composition of a cell will vary in time depending on the model of substrate assimilation (zero-order vs. first-order kinetics), the substrate assimilation rate, cell biomass, and the isotopic composition of the substrate. We specifically analyze how cell division during the incubation impacts the predicted isotopic composition of a cell and how the isotopic compositions will vary among cells within a population depending on the degree of cell cycle synchronicity. Based on these theoretical analyses, we provide nanoSIMS users with step-by-step guidelines for calculating substrate assimilation rates including formulas and their underlying assumptions. We also identify key biological factors that can introduce variability into the calculated rates, specifically highlighting the previously unrecognized role of storage inclusions and their impact on interpretations of intercellular variability in calculated assimilation rates.

## Materials and Methods

Our analysis is based on mass balances, which we formulate as differential equations to facilitate identification of parameters impacting the predicted isotopic composition of cells during a SIP experiment. We focus our analysis on assimilation of carbon (C) from a ^13^C-labeled carbon source. Analogous analyses can be conducted for other elements and their stable isotopes commonly used in SIP experiments (e.g., ^15^N, ^18^O). Throughout this study, we generally adhere to the accepted notation guidelines for reporting isotope enrichment data (Coplen, 2011) with a few exceptions (see Table 1 for a list of symbols and their definition used in this study).

### ^13^C Labeling of an Individual Cell

The model of C assimilation by an individual cell is mathematically formulated by a differential equation


(1) $$\frac{dC\left(t\right)}{dt}=r\left(t\right),$$

where *C* denotes the cell-specific C content (in mol C cell^–1^) and *r* denotes the cell-specific C assimilation rate (in mol C cell^–1^h^–1^). Both quantities are a function of time, as indicated by the argument (*t*). In this study, we neglect the potential effect of kinetic isotopic fractionation as it is typically minor compared to the changes in the isotopic composition of the cell due to labeled substrate assimilation during the SIP incubation. If needed, the effect can easily be included in the analysis described below. Additionally, we do not consider processes associated with cellular biomass turnover, e.g., exudation of freshly fixed C or respiration of storage compounds. Such processes could be implemented by assuming a second, “loss” term in Eq. 1. Their mathematical treatment is, however, beyond the scope of this study.

Given the rate of increase in the total C (i.e., ^12^*C* + ^13^*C*) content of the cell, *r*, the ^13^C content of the cell will increase at a rate *r* multiplied by the probability that the assimilated C atom is ^13^C. This probability is equal to the ^13^C atom fraction of the carbon source, denoted as *x*_*S*_. Consequently, the ^13^C content of the cell is described by a differential equation


(2) dC13(t)dt=r(t)⋅xS.

To make our analysis more general, we assume that the assimilated C may originate from two sources: (1) the target C source, which is isotopically labeled by the experimenter by the addition of a naturally less abundant stable isotope (e.g., ^13^C), and (2) an alternative C source, which only contains the natural abundance of ^13^C and may be present and assimilated by the cell without experimenter’s knowledge. For example, ^13^C-labeled dissolved inorganic carbon (DIC) is a common target C source and unlabeled dissolved organic carbon (DOC) could be an alternative C source. Assuming that C assimilations from the target and alternative sources are independent of each other, the total C and ^13^C contents of the cell will vary with time according to differential equations


(3) $$\frac{dC\left(t\right)}{dt}=r_{tar}\left(t\right)+r_{alt}\left(t\right),$$

(4) dC13(t)dt=rtar(t)⋅xS,tar+ralt(t)⋅xS,alt,

where subscripts “*tar”* and “*alt”* refer to the target and alternative C source, respectively.

Although in some SIP experiments the isotopic composition of the C sources may vary in time (e.g., Schreiber et al., 2016; Geerlings et al., 2020), we consider hereafter the more common situation that *x*_*S,tar*_ and *x*_*S,alt*_ are time-independent. We make this assumption because our aim is to derive simple formulas to calculate the substrate assimilation rate based on the isotopic composition of the cell. A more advanced analysis describing SIP experiments with time-dependent substrate labeling is provided in Supplementary Methods, Section “Modeling Cellular Assimilation of Substrates With Time-Dependent Isotopic Composition,” and in Supplementary Figures 1,2. Considering this assumption, we denote the total cell-specific C assimilation rate as *r = r_*tar*_ + r_*alt*_*, and the fraction of C assimilated by the cell from the target source as *f*_*tar*_ = *r*_*tar*_/(*r*_*tar*_ + *r*_*alt*_), which makes Eq. 3 identical to Eq. 1 and yields the following differential equation for the cellular ^13^C content:


(5) dC13(t)dt=r(t)⋅xS,eff,

where


(6) $$x_{S,eff}=f_{tar}\cdot x_{S,tar}+\left(1-f_{tar}\right)\cdot x_{S,alt}$$

is the *effective* ^13^C atom fraction of the carbon source.

**FIGURE 1:**
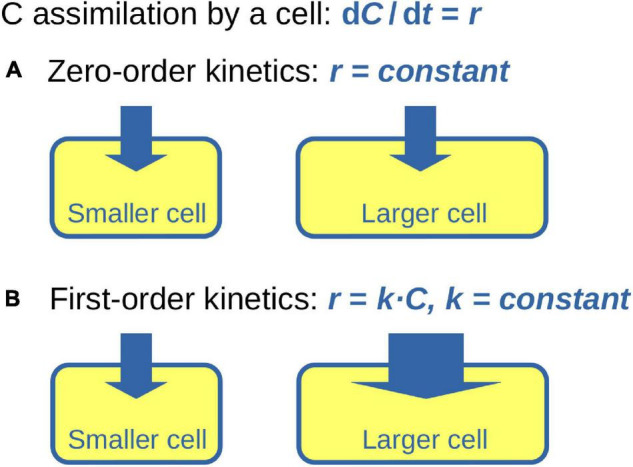
Conceptual diagram of C assimilation during cell growth. Cell is represented by a rectangle with round edges. Size of the rectangle represents C content of the cell, thickness of the arrow entering the cell represents the cell-specific rate of C assimilation, *r*. **(A)** Zero-order kinetics of C assimilation assumes that *r* is independent of the cellular C content. **(B)** First-order kinetics of C assimilation assumes that *r* increases proportionally to the instantaneous cellular C content.

To understand the consequences of C assimilation from a source characterized by *x*_*S,eff*_ for the isotopic composition of the cell requires the solutions of differential equations 1 and 5. The result depends on how *r* may or may not vary across the cell cycle. We consider two idealized scenarios: (1) *r* is constant across the cell cycle (i.e., the cell assimilates C according to zero-order kinetics), (2) *r* is linearly proportional to the instantaneous cell-specific C content, *r = k ⋅ C* (i.e., the cell assimilates C according to first-order kinetics), where *k* is constant across the cell cycle. The model of zero-order kinetics can be conceptualized as a cell whose C content increases due to the constant activity of the cell components (e.g., ribosomes, proteins) that are present at the beginning of the cell cycle and do not change in concentration over its course (Figure 1A). The model of first-order kinetics is conceptualized as described by Koch and Schaechter (1962) where the cell components are synthesized continuously and operate at constant rates throughout the cell cycle such that the ratio between these operational units and the mass of the total cell remains constant across the cell cycle (Figure 1B). Additionally, we assume that the C density of a cell, denoted hereafter as ρ (in mol C μm^–3^), is constant across the cell cycle. Variation in ρ will occur if C is assimilated into storage inclusions (e.g., polysaccharide or lipid inclusions) or if C storage inclusions are catabolized for respiration or the synthesis of other cell components during certain periods of the cell cycle. Effects of these variations are qualitatively examined in the Discussion.

#### Zero-Order Kinetics of C Assimilation

Assuming constant *r*, solutions to the differential equations 1 and 5 are readily found as


(7) $$C\left(t\right)=C_{i}+r\cdot t,$$

(8) C13(t)=Ci⋅xi+r⋅xS,eff⋅t,

where *C*_*i*_ and *x*_*i*_ denote, respectively, the C content and ^13^C atom fraction of the cell at the time-point when the SIP experiment with the labeled substrate was initiated (i.e., at *t* = 0). Thus, zero-order kinetics of C assimilation corresponds to a linear increase in cell biomass.

Equations 7-8 imply that the ^13^C atom fraction of the cell, defined as *x* = ^13^*C*/(^12^*C+*^13^*C*), increases in time according to


(9) x(t)=C13(t)C12(t)+C13(t)=C13(t)C(t)=Ci⋅xi+r⋅xS,eff⋅tCi+r⋅t.

To simplify this function, we define the *source-normalized excess ^13^C atom fraction* as


(10) $$x_{S}^{E}\left(t\right)\equiv \frac{x\left(t\right)-x_{i}}{x_{S,eff}-x_{i}}.$$

This is a convenient quantity for describing the isotopic composition of the cell because it increases from 0 to 1 as the cell increases its ^ 13^C atom fraction from *x*_*i*_ towards *x*_*S,eff*_ (see Supplementary Methods, Section “Relationships between $x_{S}^{E}$, *X*_*net*_, *Fx*_*net*_ and *K*_*A*_,” for a relationship between $x_{S}^{E}$ and the quantities *Fx*_*net*_, *K*_*A*_ and *X*_*net*_ introduced previously by other authors). Upon rearrangement, this definition combined with Eq. 9 yields


(11) $$x_{S}^{E}\left(t\right)=\frac{r\cdot t}{C_{i}+r\cdot t}.$$

**FIGURE 2:**
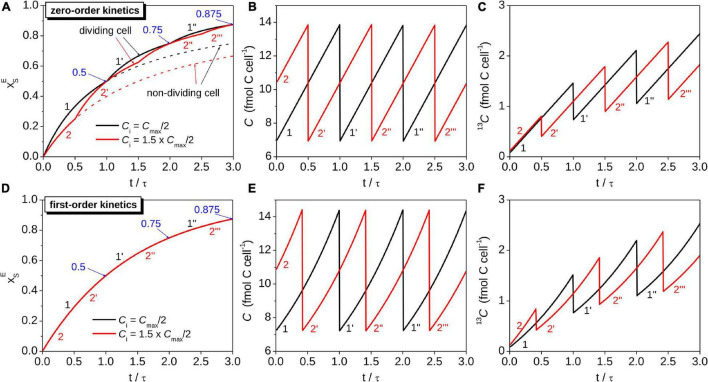
Simulations of the time-dependence of the isotopic composition and C content in cells assimilating C according to different models. Shown are the source-normalized excess ^13^C atom fraction (panels **A,D**), total C content (panels **B,E**), and ^ 13^C content (panels **C,F**) for two cells with different initial C contents (C_*i*_ = C_*max*_/2 for cell 1, C_*i*_ = 1.5⋅*C*_*max*_/2 for cell 2). Note that cells divide when their total C content reaches C_*max*_. For comparison, results calculated based on a model that ignores cell division are shown by dashed lines in panel **(A)**. The number of primes behind the digit 1 and 2 indicates the generation of the daughter cells corresponding to the original mother cells. The cells assimilating C with zero-order kinetics had equal cell-specific C assimilation rates, *r*, while the cells assimilating C with first-order kinetics had equal carbon-specific C assimilation rates, *k*. These rates were related as *k* = *r*/⟨*C*⟩, where ⟨*C*⟩ was the same for both cell types. In all panels the time was normalized by the doubling time τ, calculated as τ = *C*_*max*_/(2⋅*r*) and τ = *ln*(2)/*k* for the cells assimilating C with zero-order and first-order kinetics, respectively. Blue arrows with numbers at *t* = *n*⋅τ indicate $x_{S}^{E}=1-{\left(1/2\right)}^{n}$, where *n* = 1, 2, etc. Simulations were performed for *x*_*i*_ = 0.011 and for hypothetical values of *x*_*S*,*eff*_ = 0.2, ⟨*C*⟩ = 10 fmol C cell^–1^ and *r* = 1.35 fmol C cell^–1^ h^–1^. These values correspond to *k* = 0.135 h^–1^, τ≈ 5.1 h, and C_*max*_ ≈ 13.9 and 14.4 fmol C cell^–1^ for the cells assimilating C with zero-order and first-order kinetics, respectively.

The next step is to account for cell division. Specifically, we assume binary division, in which the cell divides into two identical cells when its C content reaches some critical value *C*_*max*_ (Collins and Richmond, 1962; Koch, 1966; Westfall and Levin, 2017). Hence, an important assumption underlying the models in this study is that cells are not starving, a condition that is commonly associated with accumulation of storage products with no regular cell division. Thus, equations 7-11 correctly describe the C content and ^ 13^C atom fraction of a cell if, and only if, *C*(*t*) is in the interval between *C*_*max*_/2 and *C*_*max*_. When *C*(*t*) in equations 7-11 reaches *C*_*max*_, it must be reset to *C*_*max*_/2. As a result, ^13^C labeling of the cell, described by the parameter $x_{S}^{E}$, will vary in time following a “zig-zag” pattern that depends on *C*_*i*_, *C*_*max*_, and *r* (see Results, Figure 2A). Hereafter, we denote the function describing this pattern as *Z*(*t*, *C*_*i*_, *C*_*max*_, *r*). The consequences of the zig-zag pattern on the resultant substrate assimilation rate are discussed in Results. Note that although zero-order kinetics of C assimilation implies a linear increase in the biomass of an individual cell (Eq. 7), binary cell division of such cells yields a population growing exponentially in cell number and total cell biomass over time.

#### First-Order Kinetics of C Assimilation

Under first-order kinetics, *r* is linearly proportional to the instantaneous cell-specific C content, i.e., *r*(*t*) = *k*⋅*C*(*t*), where *k* is a constant. The parameter *k* is commonly referred to as the growth rate constant (in h^–1^) and represents the carbon-specific rate of C assimilation [in mol C (mol C)^–1^ h^–1^]. Substituting this rate expression into Eq. 1, we obtain for the cell-specific C content


(12) $$C\left(t\right)=C_{i}\cdot e^{k\cdot t}.$$

where *C*_*i*_ is the initial C content of the cell. Thus, first-order kinetics of C assimilation corresponds to an exponentially growing cell. Note that in some studies (e.g., Martínez-Pérez et al., 2016) exponential cell growth is assumed to be described by a function 2^*Growthrate*⋅*t*^ rather than *e*^*k*⋅*t*^. These descriptions are equivalent when “*Growthrate”* is calculated as *k*/ln(2). The corresponding doubling time, τ, is calculated by considering that *C*(τ) = 2⋅*C*_*i*_, which yields a formula τ = ln(2)/*k* = 1/*Growthrate*.

To evaluate the dynamic of the ^13^C atom fraction (*x*) for an exponentially growing cell, we start from the definition of *x* and apply the quotient rule to evaluate *dx*/*dt*. Then, we use equations 1, 5 and 12 yielding the following differential equation for *x*:


(13) $$\frac{dx}{dt}=-k\cdot \left[x-x_{S,eff}\right].$$

The solution to this differential equation is


(14) $$x\left(t\right)=x_{S,eff}-\left[x_{S,eff}-x_{i}\right]\cdot e^{-k\cdot t}.$$

Using the definition in Eq. 10, this function is simplified to


(15) $$x_{S}^{E}\left(t\right)=1-e^{-k\cdot t}.$$

when the isotopic composition of the cell is described by $x_{S}^{E}$ instead of *x*. Thus, ^13^C labeling of a cell assimilating C according to first-order kinetics is described by one minus an exponential function of time.

To account for cell division, we again assume that the cell divides into two identical cells when its C content reaches some critical value *C*_*max*_. Thus, when *C*(*t*) described by Eq. 12 reaches *C*_*max*_, it must be reset to *C*_*max*_/2. In contrast to cell-specific C assimilation modeled by zero-order kinetics, this reset in *C*(*t*) due to cell division does *not* affect the time-dependence of the isotopic composition of the cell assimilating C according to first-order kinetics. This is because the assimilation rate decreases by half as the cell divides, hence the ratio of ^13^C uptake to ^12^C present in the cell remains the same. This insight also emerges directly from the differential equation 13, which shows that *x*, and hence also $x_{S}^{E}$, is independent of *C*_*i*_ (see Eq. 14-15).

### ^13^C Labeling in a Population of Cells

Here, we expand our analysis from the single cell view to a population of cells with varying degrees of cell cycle synchronization. Our aim is to reveal how the predicted isotopic enrichment varies within a population of cells assimilating C at equal rates and how this variability depends on the model used to describe C assimilation by the cell and the synchronization of the cell cycles among cells. Note that regardless of whether C assimilation is modeled by zero- or first-order kinetics, binary division at the end of each cell cycle causes the population to grow exponentially. Moreover, the population doubling time, τ, is related to the C assimilation rate according to τ = *C*_*max*_/(2⋅*r*) and τ=(*ln* 2)/*k* for the models assuming zero-order and first-order kinetics of C assimilation, respectively.

#### Zero-Order Kinetics of C Assimilation in a Population

First, we consider zero-order kinetics of C assimilation by individual cells. If cell cycles in a population are not synchronized, *C* at a given point in time varies among cells between *C*_*max*_/2 and *C*_*max*_ (Collins and Richmond, 1962; Koch, 1966). Thus, because of the dependence of the *Z* function on *C*_*i*_ (see above, Section “Zero-order Kinetics of C Assimilation”), individual cells will have different $x_{S}^{E}$ depending on their *C*_*i*_ even if they assimilate C at the same rate *r*. We denote the probability density function (PDF) describing the distribution of *C*_*i*_ and $x_{S}^{E}$ among cells by θ and *ζ*, respectively, and first consider perfectly unsynchronized and then partially synchronized cells. In both cases we assume that the critical C content where each cell divides, *C*_*max*_, is equal for all cells.

As shown by Koch (1966), the PDF for perfectly unsynchronized cells is


(16) $$\theta \left(m\right)=\frac{8\cdot ln\left(2\right)}{C_{max}}\cdot e^{-\frac{2\cdot m\cdot ln\left(2\right)}{C_{max}}}$$

for *m* in the interval *C*_*max*_/2 ≤ *m* ≤ *C*_*max*_, and it is equal to zero for *m* outside this interval. For these cells, the C content of the average cell in the population, which is equivalent to the C content averaged over the cell cycle, is related to the critical C content *C*_*max*_ by the following formula (Koch, 1966):


(17) $$\left\langle C\right\rangle =\int_{0}^{\infty }m\cdot \theta \left(m\right)dm=\frac{C_{max}}{2\cdot ln\left(2\right)}\approx 0.72\cdot C_{max}.$$

Furthermore, both θ(*m*) and ⟨*C*⟩ are time independent as the population grows.

For partially synchronized cells, θ can take various forms. Here, we assume θ to be based on a Gaussian function. However, because θ is non-zero within the interval between *C*_*max*_/2 and *C*_*max*_, the tails of the Gaussian function reaching outside this interval must be constrained within this interval with a factor that accounts for binary cell division (Supplementary Figure 3). This consideration yields


(18) $$\theta \left(m\right)=A\cdot \sum_{j=-\infty }^{\infty }2^{j}\cdot e^{-\frac{{\left(m+j\cdot C_{max}/2-C_{0}\right)}^{2}}{2{\left(\Delta C\right)}^{2}}}$$

for *m* in the interval *C*_*max*_/2 ≤ *m* ≤ *C*_*max*_, and zero for *m* outside this interval. In this function, *C*_0_ and Δ*C* describes the center and width of the Gaussian function, respectively (*C*_0_ must lie between *C*_*max*_/2 and *C*_*max*_), and *A* is the normalization constant such that $\int_{C_{max}/2}^{C_{max}}\theta \left(m\right)dm=1$.

We define the *degree of cell cycle synchronicity* in the population by the ratio γ = *C*_*max*_/(8⋅△*C*) (Supplementary Figure 3). Thus, a greater degree of synchronicity corresponds to a smaller Δ*C*, i.e., a narrower distribution of *C* among cells (Supplementary Figures 4A,B), and vice versa. Specifically, at the limits of △*C*→0 or △*C*→∞, the function θ(*m*) in Eq. 18 describes populations of cells with perfectly synchronized (γ→∞) or perfectly unsynchronized (γ→0) cell cycles, respectively. In the latter case, θ(*m*) in Eq. 18 and Eq. 16 become equivalent as required (Supplementary Figure 4C). Note that if γ is large (i.e., Δ*C* ≪ *C*_*max*_) and each cell in the population grows at the same cell-specific rate *r*, Eq. 18 implies that 95% of cells divide within a time interval △*t*_95_ = 4 ⋅ △*C*/*r* (Supplementary Figure 3). Thus, for a population with cell cycles that are highly synchronized, the parameter γ is equal to the ratio between the population doubling time, τ = *C*_*max*_/(2⋅*r*) (Table 1), and the interval during which 95% of cells undergo division (i.e., γ=τ/△*t*_95_; Supplementary Figure 3).

We performed Monte-Carlo simulations in Matlab to evaluate *ζ* from θ. Specifically, a cell *j* with a random initial C content *C*_*ij*_ is selected based on the PDF θ. Then, for a given value of *r* and *t*, the isotopic composition of daughter cells originating from cell *j* is calculated as $x_{Sj}^{E}=Z\left(t,C_{ij},C_{max},r\right)$. Additionally, the number of daughter cells originating from cell *j* is calculated from the number of cell divisions as *N*_*j*_ = 2^⌊*n*_*j*_⌋^, where ⌊*n*_*j*_⌋ denotes the largest integer that is smaller than *n*_*j*_ = (*C*_*ij*_ + *r*⋅*t*)/(*C*_*max*_/2)−1. Based on the values of $x_{Sj}^{E}$ and *N*_*j*_ obtained for many random choices of *C*_*ij*_, a histogram approximating the PDF *ζ* is reconstructed (see Results, Figure 4C). Finally, the average $x_{S}^{E}$ for all cells in the population is calculated as


(19) $$\left\langle x_{S}^{E}\right\rangle =\sum_{k}x_{S,k}^{E}\cdot \zeta \left(x_{S,k}^{E}\right),$$

where the index *k* refers to the k^*th*^ bin in the histogram approximating *ζ*. Note that both the function *ζ* and the average value $\left\langle x_{S}^{E}\right\rangle$ depend on time, *t*, although this dependence was omitted in this expression to simplify notation.

#### First-Order Kinetics of C Assimilation in a Population

Equations 14-15 show that isotopic composition of a cell assimilating C according to first-order kinetics is independent of *C*_*i*_. Thus, in contrast to a cell assimilating C according to zero-order kinetics, the average $x_{S}^{E}$ for the population of cells assimilating C by first-order kinetics is described by the same equation as for an individual cell (Eq. 15), and the variance among cells is zero.

## Results

### Modeling ^13^C Labeling in Cells

Equations 9-11 imply that ^13^C labeling of a cell assimilating C according to zero-order kinetics depends on the cell-specific rate of C assimilation, *r*, and the initial C content of the cell, *C*_*i*_. We illustrate the consequences of these dependencies by considering two cells with identical *r* but different *C*_*i*_. We assume *C*_*i*1_ = *C*_*max*_/2 for cell 1, which corresponds to a cell immediately after division (initial cell cycle stage *s*_*i1*_ = 0), and *C*_*i*2_ = 1.5⋅*C*_*max*_/2 for cell 2, which corresponds to a cell in the middle of its cell cycle (*s*_*i2*_ = 0.5; see Table 1 for the definition of *s*). Both cells then assimilate C at the same and constant rate *r* over three cell cycles (Figures 2A–C). The lower *C*_*i1*_ compared to *C*_*i2*_ causes $x_{S1}^{E}$ to initially increase faster than $x_{S2}^{E}$ (Figure 2A, *t*/τ = ≪ 0.5) and the C content of cell 2 to reach *C*_*max*_ earlier than that of cell 1. At *C*_*max*_, the rate of increase in $x_{S}^{E}$ in the two daughter cells of cell 2 abruptly increases 2-fold due to the abrupt decrease in their C content from *C*_*max*_ to *C*_*max*_/2 and an unchanged *r* (Figures 2A,B, *t*/τ = 0.5). A similar “kink” in the evolution of $x_{S}^{E}$ is observed after cell 1 divides, but it occurs later because of the lower initial C content of cell 1 (Figure 2A, *t*/τ = 1).

The $x_{S}^{E}$ values for the two cells described in Figure 2 become equal at regular time intervals. These intervals are separated by τ = *C*_*max*_/(2⋅*r*), i.e., the time needed for each cell to double its C content (Figure 2A). This result is expected because the total C content of the daughter cells at time-points *t* = *n*⋅τ (*n* = 1, 2, …) will be the same as the total C content of the original mother cell at *t* = 0 (Figures 2B,C). Hence, the zero-order kinetics model of C assimilation yields $x_{S}^{E}=1-{\left(1/2\right)}^{n}$ at time-points *t* = *n*⋅τ irrespective of the value of *C*_*i*_ (Figure 2A).

The time-dependence of $x_{S}^{E}$ in a cell assimilating C according to first-order kinetics is described by a function that is independent of *C*_*i*_ (Eq. 15). Setting the initial C contents of two cells to *C*_*max*_/2 and 1.5⋅*C*_*max*_/2 leads to different time-dependences of the total C and ^ 13^C contents for each cell (Figures 2E,F). However, the increase in $x_{S}^{E}$ over time is the same for both cells (Figure 2D). Thus, cell division and the cell’s initial C content do not influence how the cell’s isotopic composition varies in time if C assimilation can be adequately captured by the first-order kinetics model.

**FIGURE 3:**
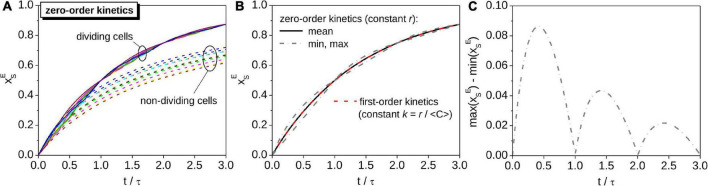
Monte-Carlo simulations of the time-dependence of $x_{S}^{E}$ in a population of cells with perfectly unsynchronized cell cycles. All cells assimilate C at the same cell-specific rate, *r*. **(A)** Time-dependence of $x_{S}^{E}$ in cells randomly selected from the population (different colors correspond to cells with different initial C content). $x_{S}^{E}$ for a dividing cell follows a pattern described by the zig-zag function Z (see Figure 2). For comparison, results calculated based on a model that ignores cell division are shown by dashed lines. **(B)** Time-dependence of $x_{S}^{E}$ for the average cell assimilating C with zero-order kinetics (black solid line), and for a cell that assimilates C with first-order kinetics with the carbon-specific rate *k* = *r*/⟨*C*⟩ (red dashed line overlapping the black solid line). Gray dash-dotted lines show the minimum and maximum values of $x_{S}^{E}$ for cells assimilating C with zero-order kinetics. The corresponding width of the interval between the minimum and maximum values of $x_{S}^{E}$ is shown in panel **(C)**. Simulations were performed for the same values of ⟨*C*⟩ and *r* as those shown in Figure 2.

While $x_{S}^{E}$ of an individual cell modeled by zero-order kinetics is described by a function with abruptly changing time-derivative (Figures 2A, 3A), $x_{S}^{E}$ for the *average* cell representing its population is described by a smooth function. If cell cycles in the population are perfectly unsynchronized, the function is identical to the function describing $x_{S}^{E}$ of a cell modeled by first-order kinetics with *k* = *r*/⟨*C*⟩, where ⟨*C*⟩ is the average C content of the cells across the cell cycle (compare solid and dashed lines in Figure 3B). Thus, for an average cell in a population with perfectly unsynchronized cell cycles, both the C content *and* ^13^C labeling behave in the same way regardless of whether their C assimilation is described by zero-order or first-order kinetics. This important result underpins the procedure for calculating rates of C assimilation in cells based on nanoSIMS data (see next section).

**FIGURE 4:**
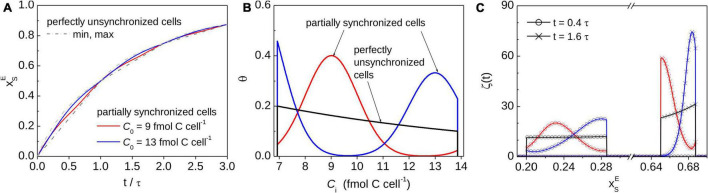
Monte-Carlo simulations of the time-dependence of $x_{S}^{E}$ in a population of cells with partially synchronized cell cycles. All cells assimilated C at the same cell-specific rate, *r*. Simulations were performed for the same values of ⟨*C*⟩ and *r* as those shown in Figure 2. The distribution of C_*i*_ among cells is described by Eq. 18 with C_0_ = 9 fmol C cell^–1^ (red) or C_0_ = 13 fmol C cell^–1^ (blue) and the degree of synchronicity γ = 1.73 (corresponding to ΔC = 1 fmol C cell^–1^, both cases). Results for perfectly unsynchronized cells (γ = 0) are shown for comparison (black). **(A)** Time-dependence of the average $x_{S}^{E}$. Dash-dotted lines show the minimum and maximum values of $x_{S}^{E}$ for a population of perfectly unsynchronized cells (reproduced from Figure 3B). **(B)** PDF describing the distribution of C_*i*_ among cells. **(C)** PDF describing the distribution of $x_{S}^{E}$ among cells, shown at two time-points (0.4 τ and 1.6 τ, where τ is the population doubling time; see legend). Colors and symbols in panels **(A–C)** correspond to each other.

If cell cycles in a population are partially synchronized (as is typical, e.g., for photoautotrophs that tune cell division according to day-night cycles), the average $x_{S}^{E}$ predicted by the zero-order kinetics model follows a zig-zag pattern like that describing $x_{S}^{E}$ for a single cell (Figure 4A). This behavior is a result of the variation of the probability density function *ζ* becoming increasingly pronounced with the increasing degree of cell cycle synchronicity (Figure 4C). However, the interval of $x_{S}^{E}$ where *ζ* is non-zero is independent of the degree of cell cycle synchronicity (Figure 4C). Thus, the value of $x_{S}^{E}$ for *any* cell from a population with partially synchronized cell cycles, including the cell representing the average of the population, always lies within the interval defined by the minimum and maximum value of $x_{S}^{E}$ determined for cells with perfectly unsynchronized cell cycles (Figures 4A,C). Note that the width of this interval varies in time following a wax-and-wane pattern oscillating between zero and a maximum value that progressively decreases with time (Figure 3C). This pattern is a consequence of binary division and the assumption that the critical C content when cells divide, *C*_*max*_, is equal among cells.

Together, these results show that cell division causes the time-dependence of $x_{S}^{E}$ in a cell modeled by zero-order kinetics to follow a zig-zag pattern that closely follows the exponential function in Eq. 15 describing the time-dependence of $x_{S}^{E}$ in a cell modeled by first-order kinetics (Figures 2A, 3A,B, 4A). In contrast, the assumption of zero-order kinetics of C assimilation with no accounting for cell division leads to a dramatically different pattern, where $x_{S}^{E}$ approaches 1 at a much slower rate and the variability among cells, caused for example by inter-cellular heterogeneity in the C content at the time of the labeled substrate addition, remains pronounced over longer time scales (compare dashed and solid lines in Figure 3A).

### Calculating Rates of Cellular C Assimilation

**FIGURE 5:**
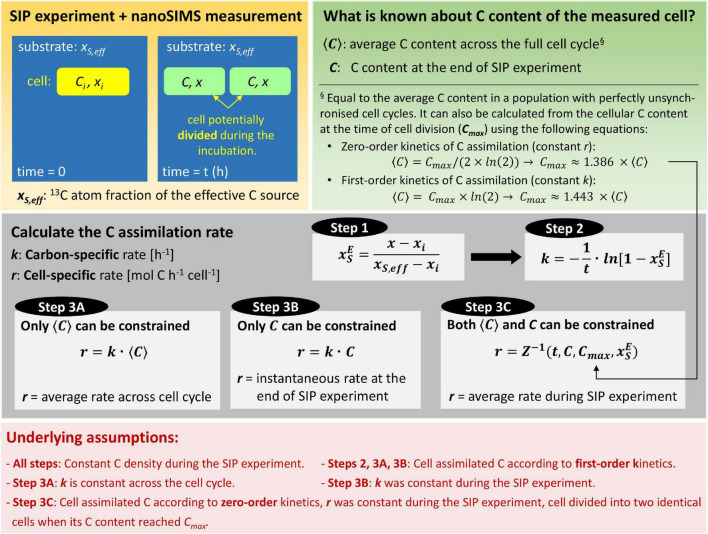
Flowchart for quantifying C assimilation rates in single cells. First, cells are incubated with a ^13^C-labeled substrate and their ^13^C atom fractions, *x*, are measured by nanoSIMS. Second, the C content of the cells is constrained. Rate calculation then proceeds in three steps: (1) convert *x* to $x_{S}^{E}$ to account for the isotopic composition of the substrate, (2) calculate the carbon-specific C assimilation rate, *k* (this rate is independent of the C content of the cell), and (3) calculate the cell-specific C assimilation rate, *r*. Depending on how well the C content of the measured cell can be constrained, the third step proceeds either by using the value of *k* from step 2 (Approach A and B) or using the value of $x_{S}^{E}$ from step 1 and the inverse of the zig-zag function, Z^–1^ (Approach C). The calculated *r* then represents the average rate across the cell cycle (Approach A), the instantaneous rate at the end of the SIP experiment (Approach B), or the average rate during the SIP experiment (Approach C). Steps 2 and 3 consider that the measured cell may be a product of a cell that divided during the incubation. The calculation approaches are implemented in a Matlab script available via GitHub (https://github.com/lpolerecky/LARS).

Using the findings presented above, we suggest a three-step procedure for calculating the cellular rates of C assimilation based on ^13^C atom fractions measured by nanoSIMS (Figure 5). We emphasize the underlying assumptions of each step to provide the foundations of the formulas. Numerical implementation of the calculation steps, including the calculation of the best estimate and uncertainty of the rates, is provided as a Matlab script that can be interfaced with experimental data organized in a spreadsheet. The script is available at https://github.com/lpolerecky/LARS. Previous studies documented that isotopic composition of cells can be significantly affected by label loss or dilution associated with common techniques of sample preparation for nanoSIMS analysis including chemical fixation, dehydration and resin embedding (Musat et al., 2012; Hoppe et al., 2013; Loussert-Fonta et al., 2020). If needed, these effects can be corrected for as previously described (Stryhanyuk et al., 2018) before applying the steps described below to calculate assimilation rates.

Step 1 converts the measured ^13^C atom fraction of the cell, *x*, to the source-normalized excess ^13^C atom fraction, $x_{S}^{E}$ (Eq. 10). This step accounts for variability in ^13^C labeling of the cell due to the ^13^C-labeled source, *x*_*S,eff*_ (see Eq. 6). Quality results require that *x*_*S,eff*_ be well constrained by the experimental set-up, such as by direct measurement. The initial ^13^C atom fraction of the cell, *x*_*i*_, is obtained by nanoSIMS measurements of control (unlabeled) cells that were prepared in the same manner as treated (labeled) cells.

Step 2 calculates the carbon-specific rate of C assimilation, *k*, according to


(20) $$k=-\frac{1}{t}\cdot ln\left(1-x_{S}^{E}\right),$$

where $x_{S}^{E}$ is obtained in Step 1. This calculation accounts for variability in ^13^C labeling of the cell due to the incubation time (Eq. 15). The underlying assumption of this step is that the measured cell assimilated C during the SIP incubation according to first-order kinetics or, equivalently, that the measured cell represents the average cell in a population that assimilated C according to zero-order kinetics, had the same cell-specific rate *r*, and had perfectly unsynchronized cell cycles (Figure 3B). Importantly, this step accounts for the possibility that the measured cell is the product of cell division that occurred during the SIP incubation.

Step 3 calculates the cell-specific rate of C assimilation, *r*. Because *r* is an absolute measure of the C assimilation rate (mol C cell^–1^ h^–1^), its accuracy depends on the knowledge of the C content of the measured cell (Supplementary Methods, Section “Estimating Cellular C Content”). There are three approaches to calculate *r*. Approach A applies when the C content of the measured cell is not known precisely but can be approximated by the average C content of the cell over a cell cycle, ⟨*C*⟩ (Figure 5, Step 3A). In this case, *r* is calculated according to


(21) r=k⋅⟨C⟩

using the value of *k* obtained in Step 2. Approach B applies when only the C content of the measured cell, *C*, but not the average C content ⟨*C*⟩, can be constrained (Figure 5, Step 3B). In this case, *r* is calculated according to


(22) r=k⋅C

using the value of *k* obtained in Step 2. Finally, Approach C applies when both *C and* ⟨*C*⟩ can be constrained (Figure 5, Step 3C). In this case, *r* is calculated according to


(23) $$r=Z^{-1}\left(t,C,C_{max},x_{S}^{E}\right)$$

using the value of $x_{S}^{E}$ obtained in Step 1. Here, *Z*^−1^ is the inverse of the zig-zag function introduced above (Figure 2A), and *C*_*max*_ is calculated from ⟨*C*⟩ using Eq. 17. We emphasize that all three approaches account for the possibility that the measured cell is the product of cell division that occurred during the SIP incubation.

The assumption underlying Approaches A and B is that the measured cell assimilated C according to first-order kinetics (i.e., at a constant *k*) during the SIP incubation. Because ⟨*C*⟩ in Eq. 21 is the average C content of the measured cell across the cell cycle, *r* calculated by Approach A represents the *average* cell-specific rate across the cell cycle. Thus, inherent to Approach A is the assumption that the measured cell assimilated C according to first-order kinetics across the entire cell cycle and with the same value of *k* as determined from the SIP experiment (i.e., by Eq. 20). In contrast, *r* calculated by Approach B represents the *instantaneous* cell-specific C assimilation rate at the time of sampling. Note that the assumption of first-order kinetics implies that *r* would have been changing during the SIP experiment (because *r* = *k ⋅ C*, where *k* is constant, and *C* is time-dependent; Figure 2E). Thus, *r* calculated by Approach B represents a rate at a specific time point during the cell cycle of the measured cell, namely at the end of the SIP experiment, when the C content reached the value of *C* used in Eq. 22.

The assumption underlying Approach C is that the measured cell assimilated C according to zero-order kinetics (i.e., at a constant *r*) during the SIP incubation and that the C content across the cell cycle varies strictly between the critical values of *C*_*max*_/2 and *C*_*max*_. Because this approach accounts for the possibility that the measured cell is the product of cell division that occurred during the SIP incubation, *r* calculated by Approach C represents the average cell-specific rate during the SIP incubation. Note that because Approach C makes use of both *C* and **⟨*C*⟩**, it allows the reconstruction of the cell cycle stage of the measured cell. Thus, if the SIP experiment is conducted over a time interval that is short compared with the doubling time of cells in a population, approach C can potentially reveal how the cell-specific rates vary across the cell cycle.

### Application to Hypothetical Data

To illustrate the utility of the procedure described above, we calculated C assimilation rates based on hypothetical data from cells incubated with a ^ 13^C-labeled C source (*x*_*S,eff*_ = 0.1) for 2 hours. Measured cells had low, intermediate, and high levels of ^13^C labeling (*x*_1_ = 0.02, *x*_2_ = 0.033, *x*_3_ = 0.09). Using Steps 1 and 2, these values yield carbon-specific assimilation rates of *k*_1_ = 0.053 h^–1^, *k*_2_ = 0.14 h^–1^, and *k*_3_ = 1.1 h^–1^, which correspond to doubling times of about τ_1_ = 13 h, τ_2_ = 4.9 h, and τ_3_ = 0.63 h, respectively.

**FIGURE 6:**
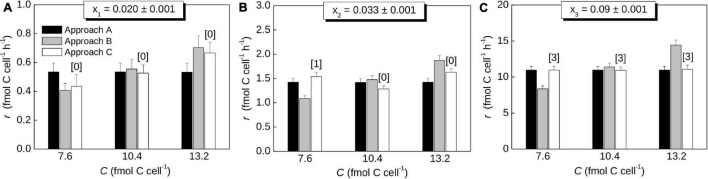
Cell-specific rates of C assimilation in cells determined using hypothetical data. The rates were calculated using the procedure described in the text (Steps 1-3) and using approaches A, B and C for Step 3. Each panel **(A–C)** corresponds to a different value of the ^13^C atom fraction of the cell, *x*, as shown above the graph. The rates represent the average rate across the cell cycle (Approach A), the instantaneous rate at the end of the SIP incubation (Approach B), and the average rate during the SIP incubation (Approach C). Values were calculated for ⟨*C*⟩ = 10 fmol C cell^–1^, *x*_*S*,*eff*_ = 0.1, *t* = 2 h, and three values of the C content of the measured cell (x-axis), each determined with the precision of 0.1 fmol. Numbers in brackets indicate how many times the measured cell divided during the incubation, as determined by the algorithm implementing Approach C. Note differences in the y-scale.

To calculate the cell-specific rate, *r*, we further assumed three values for the C content of the measured cell (*C* = 7.6, 10.4 and 13.2 fmol), each quantified with the same analytical precision of Δ*C* = 0.1 fmol, and an average C content of ⟨*C*⟩ = 10 fmol (which corresponds to the critical C content of *C_*max*_* = 13.9 fmol; Eq. 17). Figure 6 shows that *r* values predicted using Approach A do not depend on the C content of the measured cell (black bars), whereas they increase proportionally to the C content when calculated using Approach B (gray bars). This pattern is expected from Eqs. 21 and 22. In contrast, *r* calculated by Approach C (open bars) does not necessarily vary monotonously with the C content of the measured cell. This occurs if the number of divisions during the SIP incubation differs between cells with the same ^13^C labeling but with different C contents at the end of the incubation. For example, the cell with *C* = 7.6 fmol divided once, whereas the cell with *C* = 10.4 fmol did not divide during the SIP incubation (Figure 6B, open bars). Thus, the first cell needs to assimilate C more rapidly than the second cell so that both reach the same ^13^C labeling at the end of the incubation. Differences between the *r* values calculated by Approach A and C tend to diminish with increasing ^13^C labeling of the cells (compare black and open bars in Figures 6A–C). This pattern emerges because the difference between the first-order and zero-order models of C assimilation decreases with the number of cell divisions during the incubation and thus with the increasing extent of ^13^C labeling (Figure 3).

## Discussion

This work uses mathematical modeling to identify and explicitly formulate fundamental assumptions underpinning calculations of substrate assimilation rates into cells based on SIP-nanoSIMS data. The 3-step approach to calculate substrate assimilation rates presented in this research evaluates the impact of the model of C assimilation by a cell (zero-order or first-order kinetics), cell division, and the carbon content of the cell measured by nanoSIMS on the calculated rate. Below, we summarize the key assumptions underlying this 3-step approach and compare it to previously published approaches for determining rates of substrate assimilation. Then, we discuss how the rates evaluated in single cells can be upscaled toward a cell population. Finally, motivated by results from a recent nanoSIMS study on diazotrophic cyanobacteria (Polerecky et al., 2021), we identify key sources for the variation in calculated assimilation rates typically observed among cells of the same species within a population that have not been previously discussed.

### Summary of Assumptions Used to Calculate Assimilation Rates

Important assumptions underlying the calculations described in this study are: (1) Catabolism or turnover of cell material, such as protein and carbohydrates, does not occur even though their half-lives may be shorter than the incubation duration. Thus, the cell-specific C assimilation rate directly reflects the increase in the cell biomass over time (Eq. 1). (2) The cell’s activity is constant during the incubation. That is, the C assimilation rates *r* and *k* in the zero-order and first-order kinetic models, respectively, are time-independent. (3) Carbon density of the cells is constant or unaffected by accumulation of storage inclusions. (4) A cell divides into two identical cells when its C content reaches a certain critical value *C*_*max*_. (5) The effect of kinetic isotope fractionation is minor and negligible. (6) Isotopic composition of the substrate, *x*_*S*,*eff*_, does not change during the incubation. Analysis of a SIP incubation with time-dependent *x*_*S*,*eff*_ is discussed in more detail in Supplementary Methods, Section “Modeling Cellular Assimilation of Substrates With Time-Dependent Isotopic Labeling.”

### Comparison With Previous Studies

Commonly used approaches for calculating substrate assimilation rates (Foster et al., 2013; Krupke et al., 2015; Svedén et al., 2015; Klawonn et al., 2016; Stryhanyuk et al., 2018; Calabrese et al., 2019; Mills et al., 2020) assume a zero-order kinetic model of substrate assimilation and do not consider that the measured cell could be a product of cell division during the SIP incubation. Additionally, they typically use an average value for the C content or the C density combined with the biovolume of the measured cell. Here we evaluate the impact of these assumptions on calculated rates.

The most common published approaches calculate *r* according to formulas equivalent to (Krupke et al., 2015; Svedén et al., 2015; Klawonn et al., 2016; Mills et al., 2020; Ploug, 2021)


(24) $$r=\frac{1}{t}\cdot x_{S}^{E}\cdot C_{f},$$

or (Stryhanyuk et al., 2018; Calabrese et al., 2019)


(25) $$r=\frac{1}{t}\cdot \frac{x_{S}^{E}}{1-x_{S}^{E}}\cdot C_{i}.$$

In these formulas, *C*_*i*_ and *C*_*f*_ denote the C content of the measured cell at the beginning and end of the SIP incubation, respectively. Equations 24 and 25 are equivalent and follow directly from the mass balance of a *non-dividing* cell. Equation 24 is obtained by defining the cell-specific C assimilation rate as *r* = *C*_*a*_/*t*, where *C*_*a*_ is the amount of carbon assimilated by the cell during time *t*, and by considering that $x_{S}^{E}=C_{a}/C_{f}$ for a cell that did not divide during time *t* (see Supplementary Methods, Section “Relationships between $x_{S}^{E}$, *X*_*net*_, *Fx*_*net*_ and *K*_*A*_”). Similarly, Eq. 25 derives from the same assumptions and by additionally considering that, for a cell that did not divide, *C*_*i*_ and *C*_*f*_ are related according to *C*_*f*_ = *C*_*i*_+*C*_*a*_. In summary, these equations yield the average cell-specific C assimilation rate during the SIP incubation. However, the calculated *r* is accurate only if the cell did not divide during the incubation and if *C*_*f*_ and *C*_*i*_ are stringently constrained.

Constraining *C*_*i*_ or *C*_*f*_ of a cell is not trivial even without considering cell division. Facing the practical difficulties of their direct measurement, some practitioners use ⟨*C*⟩ in place of *C*_*f*_ (e.g., Svedén et al., 2015; Olofsson et al., 2019a) or approximate *C*_*i*_ using *C*_*f*_ (Stryhanyuk et al., 2018; Calabrese et al., 2019). These substitutions, however, invalidate the approach based on Eq. 24 or 25 because the mass balance underpinning these formulas is no longer achieved.

Many studies estimate the biovolume of the measured cells from nanoSIMS images and apply literature values for the C density, or an empirical relationship between the C density and biovolume of phytoplankton cells (e.g., Verity et al., 1992; Stryhanyuk et al., 2018; Khachikyan et al., 2019), to approximate *C*_*f*_ (e.g., Foster et al., 2013; Krupke et al., 2015; Schoffelen et al., 2018; Mills et al., 2020; Trembath-Reichert et al., 2021). Although this approach may yield a well-constrained value of *C*_*f*_, one must be careful when using it in Eq. 24 to calculate *r*. Specifically, if the cell divided during the SIP incubation, the correct value of *C*_*f*_ should include the C content of the *measured* cell as well as its *sister* cell (or sister cells, if the cell divided more than once). Only then will the mass-balance requirement be fulfilled, and Eq. 24 will yield the correct value of *r* for the measured cell. This could, in principle, be achieved by including a correction factor when calculating the *cell*-specific rate using Eq. 24. Several recent studies (Schoffelen et al., 2018, 2019; Olofsson et al., 2019a,b; Ploug, 2021) have employed a factor 2 in the calculation of *carbon*-specific growth rates. However, as this latter quantity is solely determined by the cell’s isotopic composition (i.e., it does not describe absolute amounts of carbon assimilated; see Eq. 20), this operation does not correct for the carbon content of a sister cell as described above.

**FIGURE 7:**
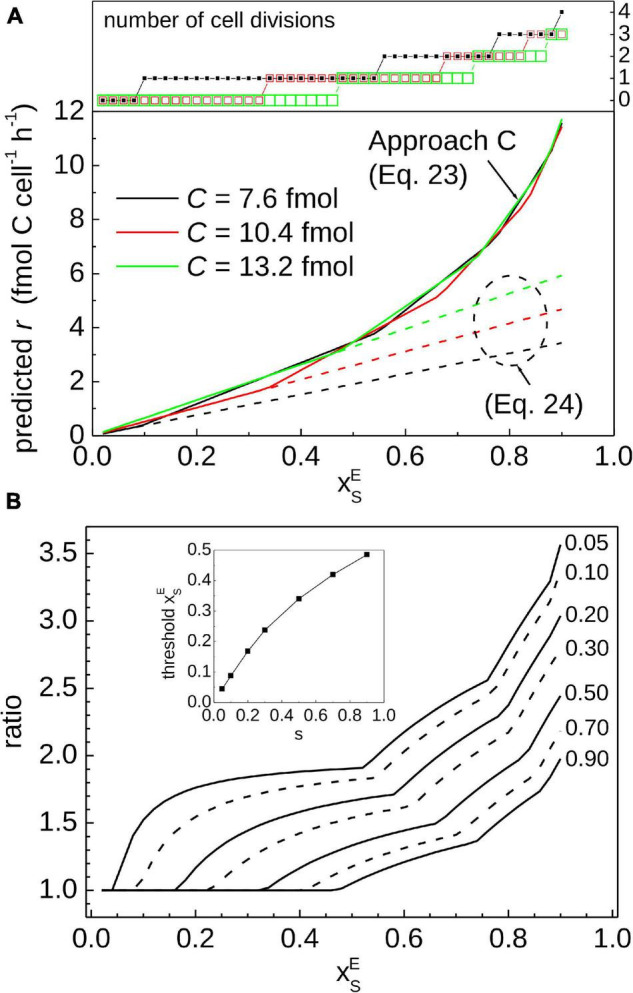
Comparison of previously published and the current approaches for calculating C assimilation rates in cells. The key difference is that our Approach C accounts for cell division, whereas cell division is not considered in the approach using Eq. 24. **(A)** Carbon-specific rates predicted from the ^13^C labeling of the measured cell, expressed as $x_{S}^{E}$. Calculations assumed ⟨*C*⟩ = 10 fmol C cell^–1^, *t* = 2 h, and three values of the cell’s C content, *C*. The values of *C* = 7.6, 10.4, and 13.2 fmol correspond to the cell cycle stage of *s* = 0.1, 0.5, and 0.9, respectively. **(B)** Ratio between the *r* value predicted by Approach C and the approach based on Eq. 24, calculated for several values describing the cell cycle stage of the measured cell (indicated by numbers next to the lines). Approach based on Eq. 24 yields lower rates compared to Approach C if the measured cell divided during the incubation [see number of divisions in the top graph in panel **(A)**]. This occurs when $x_{S}^{E}$ exceeds a threshold value that increases with the increasing cell cycle stage of the measured cell (see inset in panel **(B)**). Note that the ratio only depends on $x_{S}^{E}$ and *s* and not on the incubation time.

To illustrate quantitatively the impact of cell division on calculated *r*, we compared the results obtained using Eq. 24 and our Approach C (Eq. 23) for a range of ^13^C labeling and C content of the measured cell (Figure 7). We did not apply a correction factor to calculate *C*_*f*_ but assumed that *C*_*f*_ in Eq. 24 is the same as *C* in Eq. 23. As expected, both approaches yield the same result if the cell did not divide during the SIP incubation (see values in Figure 7 for which the number of cell divisions is equal to 0), because the function in Eq. 11, which is the basis for Eq. 24, is equal to the zig-zag function *Z* (see Figure 2A, black curve before t/τ < 1 and red curve before t/τ < 0.5). However, if the measured cell did divide during the incubation, Eq. 24 yields a lower *r* value compared to that calculated by Approach C (Figure 7). The difference between the two approaches becomes more pronounced as $x_{S}^{E}$ of the measured cell increases above a threshold value that depends on the cell cycle stage (Figure 7B). For example, the threshold value of $x_{S}^{E}$ is 0.1 if the cell cycle stage of the measured cell is 10%, but it increases to 0.33 and 0.47 for the cell cycle stage of 50% and 90%, respectively (inset in Figure 7B).

The question remains, however, how to determine whether a cell measured by nanoSIMS has divided during the SIP incubation. Tracing the history of a cell analyzed by nanoSIMS is practically impossible because nanoSIMS is a terminal measurement. The history can be reconstructed if additional information about the measured cell, namely ⟨*C*⟩, can be constrained. Constraining ⟨*C*⟩ allows evaluation of the minimum and maximum C content of the measured cell (*C*_*max*_/2 and *C*_*max*_, respectively; Eq. 17). Combining this information with the carbon content, *C*, enables estimation of the cell cycle stage of the measured cell. Combined with the value of $x_{S}^{E}$, the history of the measured cell during the SIP experiment can be reconstructed to estimate the value of *r* that accounts for cell division. This sequence of steps is implemented by our Approach C based on the inverse of the zig-zag function (Eq. 23).

We conclude that the 3-step procedure proposed in this study (Figure 5) is applicable to many situations encountered by users of SIP-nanoSIMS to estimate substrate assimilation rates in individual cells. When the C content of a measured cell, *C*, cannot be constrained, the carbon-specific assimilation rate, *k* (Eq. 20), and the corresponding doubling time, τ=(*ln*2)/*k*, is the maximal information that can be estimated from the measured isotopic composition of the cell. Constraining *C* allows estimation of the instantaneous cell-specific assimilation rate at the end of the SIP experiment (Approach B, Eq. 22), while constraining ⟨*C*⟩ allows extrapolation towards the average cell-specific assimilation rate across the cell cycle (Approach A, Eq. 21). Finally, constraining both *C* and ⟨*C*⟩ allows determination of the average cell-specific assimilation rate over the SIP experiment (Approach C, Eq. 23). Note that accounting for cell division, as done in Approach C, critically depends on the assumption that the measured cell divided during the incubation when its *C* content reached *C*_*max*_ estimated from ⟨*C*⟩. If this assumption is not valid (e.g., because the C assimilated by the cell was allocated into storage inclusions, or cell division was delayed or not binary), or cannot be verified, the instantaneous rate at the end of the SIP experiment (Eq. 22) is the maximal information about the cell-specific assimilation rate that can be estimated by combining the ^13^C labeling and C content of the measured cell.

### Extrapolation Toward a Cell Population

A frequent aim of SIP-nanoSIMS measurements is to estimate a bulk C assimilation rate for a target cell population and thus evaluate its potential impact on C fluxes in the environment. This upscaling can be done by first averaging the cell-specific C assimilation rates determined for individual target cells to estimate the population average, ⟨*r*⟩, and then multiplying ⟨*r*⟩ by the total target cell abundance in the environment. When calculating the assimilation rates for individual cells, Approach A or B is recommended if cell cycles in the target population are perfectly unsynchronized, whereas Approach C is recommended for partially synchronized cells. Because these approaches account for cell division, ⟨*r*⟩ estimated in this way will reflect the population average across a broad range of incubation times (Supplementary Figure 5), which may be important when using SIP-nanoSIMS to study environmental samples where the growth rates of cells are *a-priori* unknown. Averaging of cell-specific rates calculated using Eq. 24 is not recommended if the expectation is that a significant number of target cells divided during the incubation, because this approach would underestimate ⟨*r*⟩ (Supplementary Methods, Section “Simulating SIP Incubation,” Supplementary Figure 5). Note that the precision and accuracy of the estimated bulk assimilation rate will still depend on the number of target cells measured by nanoSIMS, i.e., on the quality of sampling of the target population.

### Interpretation of Calculated Rates and Their Variability Among Cells

Typically, ^13^C atom fractions obtained by nanoSIMS measurements vary among cells, raising the question, to what extent this variability is caused by differences in the intrinsic metabolic activities of cells probed by the SIP experiment (i.e., *r*) versus differences arising from cells at different stages of their cell cycle during the labeling interval. Additionally, we note that assimilation rates obtained in nanoSIMS studies can reflect variation not only in the targeted process (e.g., C fixation) but also in other metabolic processes and cellular characteristics (e.g., simultaneous assimilation of other external or internal C sources). In this study we identified that in addition to *r*, the sources of variability include *t*, *x*_*S,eff*_, and *C*_*i*_. Our 3-step procedure (Figure 5) accounts for some, but not all, of these sources of variability. Below, we discuss what this uncertainty implies for interpreting the calculated rates of C assimilation (*k* or *r*) and especially their variability among measured cells. We focus on C, but similar arguments can be made for other elements.

#### Influence of an Alternative C Source

The effective isotopic composition of the C source, *x*_*S,eff*_, will almost certainly be different from the isotopic composition of the target C source, *x*_*S*_, if cells assimilate carbon from an alternative source in addition to the target source. The parameter *f*_*tar*_ accounts for the influence of alternative C sources (see Materials and Methods and Table 1). Calculated *k* values will underestimate total C assimilation if an alternative C source influenced *x*_*S,eff*_ (Table 2). Thus, cell-to-cell differences in *k* may reflect inter-cellular variation in use of the target source rather than total C assimilation. Cell-specific rates, *r*, will be similarly affected as they are related to *k*.

**TABLE 2:** Simulated effect of C assimilation from two sources.

cell	*f*_*tar*_	*x*	*x*_*S,eff*_		*k* (h^–1^)	
			Correct	Assumed in calculation	Calculated	Total
1	0.5	0.0197	0.505	0.99	0.005	0.01
2	0.8	0.0255	0.802	0.99	0.008	0.01
1	0.5	0.0197	0.505	0.505	**0.01**	**0.01**
2	0.8	0.0255	0.802	0.802	**0.01**	**0.01**

*Shown are results for two cells that assimilate C with equal total carbon-specific rates of 0.01 h^–1^ over 2 h. The cells differ only in the fraction of C assimilated from the target C source, f_tar_. If C is incorrectly assumed to be assimilated from only the target source, calculated k will underestimate the total C assimilation rate (as discussed in Section Influence of an Alternative C Source). The calculated and total values will only be equal if C assimilation from the alternative C source is accounted for by using the correct value for x_S,eff_ (lines 3-4, values shown in bold). Calculated for x_S,tar_ = 0.99, x_S,alt_ = 0.011, and x_i_ = 0.011.*

#### Influence of C Storage Content

Because *k* is defined as *k* = *r*/*C*, variability in calculated *k* among cells can be caused not only by variation in the instantaneous C assimilation rate (*r*) but also by variation in the instantaneous C content (*C*). Such variations in *C* can be expected, for example, in cells that encounter starvation, i.e., they have depleted a required growth resource from the environment, or the environment has changed, such that assimilation of that resource is no longer possible. These conditions cause cells to cease exponential growth and commonly lead to the synthesis of storage compounds (e.g., polysaccharides). The variability in calculated *k* (Eq. 20) among starving cells may therefore reflect variation in storage content rather than *r*.

Similarly, cell-to-cell variability in calculated *r* needs to be interpreted with caution as it may reflect variation in storage content, rather than intrinsic cell-specific C assimilation rates, because neither Approach A, B nor C accounts for potential variation in the cell’s C content caused by storage (see the underlying assumptions in Figure 5). Specifically, *r* is calculated either directly from *k* using ⟨*C*⟩ (Approach A, Eq. 21), or the calculation relies on the assumption that the cell C density, which may be used to estimate the C content of the measured cell based on its biovolume (Supplementary Methods, Section “Estimating Cellular C Content”), is constant during the SIP experiment and equal among cells (Approach B and C, Eq. 22-23). These assumptions are unlikely to be valid in cells that metabolize storage inclusions. By accounting for the relationships between storage content and apparent assimilation rates, however, we can deduce information about storage metabolism from nanoSIMS data, as outlined in the following section.

#### Interpretation of Simultaneous ^13^C and ^15^N Labeling Experiments

For a single labeled element (e.g., ^13^C or ^15^N), the importance of storage metabolism versus assimilation of new material is difficult to resolve without additional measurements at subcellular level (e.g., TEM images of storage inclusions). On the other hand, dual labeling (i.e., simultaneous incubations with two labeled substrates such as ^13^C and ^15^N) can greatly increase informative value of the SIP experiment. By systematic consideration of the effects of storage inclusion metabolism on the element-specific rates of C and N assimilation (*k*_*C*_ and *k*_*N*_), whole-cell nanoSIMS measurements can be used to gain information not only about the acquisition of new carbon and nitrogen, but also about intracellular fluxes of these elements (Polerecky et al., 2021). Specifically, metabolic pathways leading to storage inclusion biosynthesis and catabolism can be revealed by leveraging information about the known elemental compositions of specific storage inclusions (e.g., polysaccharides or cyanophycin) and the isotopic compositions of target C and N sources in carefully designed SIP experiments.

We simulated assimilation of ^13^C- and ^15^N-labeled substrates by cells under various scenarios, including preferential incorporation of the assimilated C and N into different cell compartments (e.g., cell matrix or storage inclusions), or C and N incorporation using internally recycled C and N in addition to C and N originating from the target sources provided externally (Supplementary Methods, Section “Modeling Simultaneous Assimilation of C and N”). Subsequently, we calculated the C- and N-specific assimilation rates, *k*_*C*_ and *k*_*N*_, in these cells using the approach described above (Eq. 20).

**FIGURE 8:**
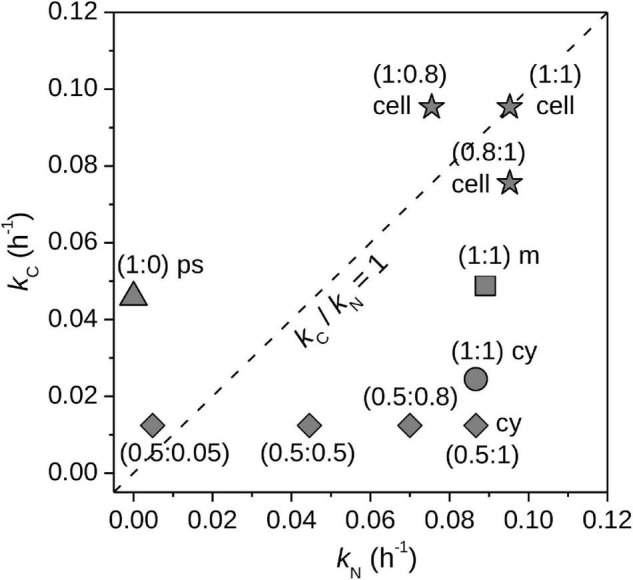
Simulations of cell-specific assimilation of C and N. Element-specific rates of C and N assimilation by whole cells, *k*_*C*_ and *k*_*N*_, calculated from hypothetical data. The first simulation (stars) shows the results for assimilated C and N that originated from two sources: labeled target sources [*x*(^13^C)_*S,tar*_ = 1, *x*(^15^N)_*S,tar*_ = 1] and unlabeled alternative sources [*x*(^13^C)_*S,alt*_ = 0.011, *x*(^15^N)_*S,alt*_ = 0.0037]. Resultant *k*_*C*_ and *k*_*N*_ values assumed assimilation of only the target sources [x(^13^C)_*S,eff*_ = 1, x(^15^N)_*S,eff*_ = 1]. Individual data-points correspond to cells that assimilated different fractions of C and N from the target sources, with corresponding *f*_*C*,*tar*_ and *f*_*N*,*tar*_ values given in parentheses (as *f*_*C*,*tar*_:*f*_*N*,*tar*_). Deviation of *k*_*C*_/*k*_*N*_ from 1 reveals assimilation of C or N from alternative sources. The second simulation shows the impacts of preferential incorporation of C and N into cyanophycin granules (cy, circle), polysaccharide inclusions (ps, triangle), and cell matrix (m, square). Cellular compartments with C:N that differ from the whole cell can cause *k*_*C*_/*k*_*N*_ to deviate from 1. The third simulation (diamonds) shows results for cyanophycin synthesis using unlabeled C and N from inside the cell (e.g., protein and polysaccharide catabolism) in addition to C and N from the external target sources. The parentheses give *f*_*C*,*tar*_:*f*_*N*,*tar*_. Use of unlabeled internal C and N pools causes the cell to assimilate C and N from sources with x(^13^C)_*S,eff*_ < 1 and x(^15^N)_*S,eff*_ < 1, whereas calculation of *k*_*C*_ and *k*_*N*_ assumed x(^13^C)_*S,eff*_ = 1 and x(^15^N)_*S,eff*_ = 1. Details of the simulations are given in Supplementary Methods, Section “Modeling Simultaneous Assimilation of C and N.”

If a cell assimilated the ^13^C- and ^15^N-labeled source substrates in a balanced way, i.e., the amounts of assimilated C and N did not change the C:N ratio of the cell, the ratio of the calculated *element*-specific rates of C and N assimilation, *k*_*C*_/*k*_*N*_, is equal to 1. Deviation of *k*_*C*_/*k*_*N*_ from 1 can arise for multiple reasons. First, the effective isotopic composition of the C or N source, *x*(^13^C)*_*S,eff*_* or *x*(^15^N)*_*S,eff*_*, differed from the isotopic composition of the target C and N source (Figure 8, stars). For example, by providing autotrophs with ^13^C-DIC (their only carbon source) and ^15^N-ammonium, a value of *k*_*C*_/*k*_*N*_ > 1 could indicate uptake of organic N compounds in addition to ammonium. Thus, deviation of *k*_*C*_/*k*_*N*_ from 1 can reveal uptake of additional substrates. A similar approach was used by Schoffelen et al. (2018) to reveal uptake of organic P in addition to inorganic P. Second, C and N assimilation did not match the C:N ratio of the whole cell. For example, ^13^C or ^15^N were incorporated into inclusions with C:N ratios that differed from that of the whole cell (Figure 8, circle, square, triangle). This can happen in cells that temporally decouple assimilation of C and N, such as in some diazotrophs, which assimilate C in the light and N mostly in the dark (e.g., Polerecky et al., 2021). Third, C or N from existing cell material (e.g., proteins, storage compounds) was mobilized into a new form. For example, protein degradation released N that was then used for cyanophycin synthesis in addition to incorporation of ^15^N from the target source (Figure 8, diamonds; see also Polerecky et al., 2021). Thus, deviation of *k*_*C*_/*k*_*N*_ from 1 can be used to identify the presence of intra-cellular C and N stores and evaluate their synthesis or mobilization without the need for direct measurement at the sub-cellular level. Notably, the assessment of the deviation of *k*_*C*_/*k*_*N*_ from 1 is solely based on the nanoSIMS measurement and does not require knowledge about the C and N content of the measured cell.

## Conclusion

This study revisited calculations of biologically relevant parameters from SIP-nanoSIMS data. We suggest a step-by-step procedure to evaluate elemental assimilation rates into whole cells, discuss key assumptions underlying the procedure, and describe factors that can introduce variability into the calculated rates.

Determining single-cell assimilation rates is challenging, as it requires proper evaluation of multiple factors that are difficult to constrain experimentally or analytically, necessitating assumptions in the calculation process. Our analysis illustrates and quantifies the consequences of some of the key assumptions, particularly focusing on assimilation kinetics (zero vs. first order), cell division, and the elemental content of the measured cell. We give guidance for interpreting the rates calculated by approaches that differ depending on knowledge of the elemental content of the measured cell and develop an approach to account for cell division in the calculation process.

We call for caution when interpreting inter-cellular variability of assimilation rates calculated from SIP-nanoSIMS data if (1) cells can assimilate elements such as C from alternative sources present in the environment in addition to the target source added externally during the SIP experiment, (2) storage inclusions are synthesized, catabolized or merely present inside the studied cells during the SIP experiment, or (3) cell divisions during the SIP experiment may be expected. Our modeling analysis further shows that for dual labeling SIP experiments (e.g., using ^13^C and ^15^N labeled substrates), deviation of *k*_*C*_/*k*_*N*_ from 1 can be used to identify the presence of intra-cellular C and N stores and evaluate their synthesis or mobilization without the need for direct measurement at the sub-cellular level.

## Data Availability Statement

The original contributions presented in the study are included in the article/Supplementary Material, further inquiries can be directed to the corresponding author/s.

## Author Contributions

LP conceived the study and performed the mathematical analysis and modeling. LP, KH, and ME wrote the manuscript with significant contributions from all other co-authors. All authors contributed with ideas that shaped the final version of the manuscript.

## Conflict of Interest

The authors declare that the research was conducted in the absence of any commercial or financial relationships that could be construed as a potential conflict of interest.

## Publisher’s Note

All claims expressed in this article are solely those of the authors and do not necessarily represent those of their affiliated organizations, or those of the publisher, the editors and the reviewers. Any product that may be evaluated in this article, or claim that may be made by its manufacturer, is not guaranteed or endorsed by the publisher.
