# Supplementary material for: Calculation and Interpretation of Substrate Assimilation Rates in Microbial Cells Based on Isotopic Composition Data Obtained by nanoSIMS
Source: Front Microbiol. 2021 Nov 30;12:621634. doi: 10.3389/fmicb.2021.621634 (PMC8670600; doi:10.3389/fmicb.2021.621634)
Supplement: Supplementary file 1 [file Data_Sheet_1.PDF]

Supplementary Material

1 Supplementary Methods

1.1 Modeling cellular assimilation of substrates with time-dependent isotopic composition

As explained in the main text, the isotopic composition of a cell assimilating C from two isotopically labeled sources can be predicted by solving differential equations 3 and 4. Those differential equations were solved using the assumption that the ^{13}C atom fraction of the C sources is constant, which is a reasonable approximation for most SIP experiments. Here, we show how the differential equations 3 and 4 are solved when the isotopic composition of the C sources varies linearly over time, as may occur in some SIP experiments (e.g., Geerlings et al. 2020). The key results of this Supplement are formulated by equations S9, S10, and S15 and illustrated in **Supplementary Figures S1** and **S2**.

To simplify notation, we use here subscripts '1' and '2', rather than 'tar' and 'alt', when referring to the target and alternative C source, respectively. Thus, we have

$$x_{S1}(t) = x_{S1,i} + K_{S1} \cdot t, \qquad (S1)$$

$$x_{S2}(t) = x_{S2,i} + K_{S2} \cdot t, \qquad (S2)$$

for the isotopic composition of source 1 and 2, where $x_{S1,i}$ and $x_{S2,i}$ denotes the initial (i.e., at $t = 0$) isotopic composition of the respective source and K_{S1} and K_{S2} (both in d^{-1}) describe the respective rate of change over time. The values of $x_{S1,i}$, $x_{S2,i}$, K_{S1}, and K_{S2} are assumed to be known. We neglect kinetic isotopic fractionation and assume that the fraction of C assimilated by the cell from the target source (see parameter f_{tar} in the main text) is constant.

1.1.1 Zero-order kinetics of C assimilation

Under the assumption of zero-order kinetics of C assimilation, both cell-specific C assimilation rates r_1 and r_2 are constant and the differential equations for the total C and ^{13}C content of a cell are written as

$$\frac{dC}{dt} = r_1 + r_2, \qquad (S3)$$

$$\frac{d^{13}C}{dt} = (x_{S1,i} + K_{S1} \cdot t) \cdot r_1 + (x_{S2,i} + K_{S2} \cdot t) \cdot r_2. \qquad (S4)$$

Differential equations S3 and S4 can be readily integrated because the functions on the right-hand side are linear. Specifically, we obtain

$$C(t) = C_i + (r_1 + r_2) \cdot t, \qquad (S5)$$

$$^{13}C(t) = {}^{13}C_i + (x_{S1,i} \cdot r_1 + x_{S2,i} \cdot r_2) \cdot t + (K_{S1} \cdot r_1 + K_{S2} \cdot r_2) \cdot \frac{t^2}{2}, \qquad (S6)$$

where C_i and ${}^{13}C_i$ are the initial (i.e., at $t = 0$) values of the total C and ^{13}C content of the cell. Thus, an explicit expression describing the isotopic composition of a cell is obtained from the definition $x(t) \equiv {}^{13}C(t)/C(t)$ using the expressions in equations S5 and S6:

$$x(t) = \frac{C_i \cdot x_i + (x_{S1,i} \cdot r_1 + x_{S2,i} \cdot r_2) \cdot t + (K_{S1} \cdot r_1 + K_{S2} \cdot r_2) \cdot \frac{t^2}{2}}{C_i + (r_1 + r_2) \cdot t}, \qquad (S7)$$

where $x_i = {}^{13}C_i/C_i$ denotes the initial isotopic composition of the cell.

A more convenient quantity describing the isotopic composition of a cell is the excess ${}^{13}C$ atom fraction, which is defined as (see Coplen, 2011)

$$x^E(t) = x(t) - x_i. \qquad (S8)$$

Combining equations S7 and S8, we obtain

$$x^E(t) = \frac{(x^E_{S1,i} \cdot r_1 + x^E_{S2,i} \cdot r_2) \cdot t + (K_{S1} \cdot r_1 + K_{S2} \cdot r_2) \cdot \frac{t^2}{2}}{C_i + (r_1 + r_2) \cdot t}, \qquad (S9)$$

where

$$x^E_{S1,i} = x_{S1,i} - x_i$$

and

$$x^E_{S2,i} = x_{S2,i} - x_i$$

describe the initial excess ${}^{13}C$ atom fraction of the target and alternative C source, respectively.

Equation S9 can be further rearranged if we define the following quantities:

- the total cell-specific assimilation rate,

$$r = r_1 + r_2,$$

- the fraction of C assimilated from the target source (source 1),

$$f_1 = \frac{r_1}{r_1 + r_2},$$

- the initial effective ${}^{13}C$ atom fraction of the sourse (analogous to $x_{S,eff}$ defined by Eq. 6 in the main text),

$$x_{S,eff,i} = f_1 \cdot x_{S1,i} + (1 - f_1) \cdot x_{S2,i},$$

- the effective rate of change of the C source,

$$K_{S,eff} = f_1 \cdot K_{S1} + (1 - f_1) \cdot K_{S2},$$

- and the initial source-normalized excess ${}^{13}C$ atom fraction (analogous to x^E_S defined by Eq. 10 in the main text),

$$x_{S,i}^{E}(t) = \frac{x(t) - x_i}{x_{S,eff,i} - x_i}.$$

Substituting these definitions into Eq. S9, we can rewrite the isotopic composition of the cell in a form that is analogous to that in Eq. 11 in the main text, but additionally accounts for isotopic compositions of the C sources that vary linearly over time:

$$x_{S,i}^{E}(t) = \left(1 + \frac{K_{S,eff}}{x_{S,eff,i}} \cdot \frac{t}{2}\right) \cdot \frac{r \cdot t}{C_i + r \cdot t}. \qquad (S10)$$

Note that expressions S9 and S10 describe the isotopic composition of a cell that does not divide during the time interval between 0 and t. Accounting for cell division is done as explained in the main text. Also note that expression S10 is equivalent to expression 11 in the main text if the isotopic composition of the two sources is time-independent (i.e., if K_{S1} and K_{S2}, and thus also $K_{S,eff}$, are equal to zero), as required.

1.1.2 First-order kinetics of C assimilation

Under the assumption of first-order kinetics of C assimilation, the cell-specific C assimilation rates are linearly proportional to the instantaneous C content of the cell: $r_1 = k_1 \cdot C$ and $r_2 = k_2 \cdot C$, where k_1 and k_2 are rate constants describing C assimilation from the respective sources.

To find an expression for the isotopic composition of a cell as a function of time, we first derive differential equations for $x(t)$ and $x^E(t)$ from differential equations 3 and 4 in the main text. We start from the definition of $x(t) \equiv {}^{13}C(t)/C(t)$, apply the rule for the derivative of a ratio of two functions, and substitute for dC/dt and $d^{13}C/dt$ from equations 3 and 4 in the main text. These steps yield[1]

$$\begin{aligned} \frac{dx}{dt} &= \frac{d}{dt}\left(\frac{{}^{13}C}{C}\right) = \frac{1}{C^2} \cdot \left(\frac{d^{13}C}{dt} \cdot C - \frac{dC}{dt} \cdot {}^{13}C\right) = \frac{1}{C} \cdot \frac{d^{13}C}{dt} - \frac{1}{C} \cdot \frac{dC}{dt} \cdot \frac{{}^{13}C}{C} \\ &= \frac{1}{C} \cdot (k_1 \cdot x_{S1} \cdot C + k_2 \cdot x_{S2} \cdot C) - \frac{1}{C} \cdot (k_1 \cdot C + k_2 \cdot C) \cdot x. \end{aligned}$$

After rearranging the intermediate result above, we obtain the following differential equation for x:

$$\frac{dx}{dt} = -k_1 \cdot (x - x_{S1}) - k_2 \cdot (x - x_{S2}). \qquad (S11)$$

This differential equation is analogous to Eq. 13 in the main text, but it accounts for C assimilation from two sources rather than one.

Now we consider that the initial composition of the cell, x_i, is a constant. Thus, for the excess ${}^{13}C$ atom fraction defined by Eq. S8, we have $dx/dt = dx^E/dt$. This equality together with Eq. S8 implies that the differential equation S11 for x can be rewritten for x^E as follows:

---

[1] Note that to simplify notation, the argument (t) indicating the time-dependence of x_{S1} and x_{S2}, as described in equations S1–S2, is omitted in the derivations leading to equations S11–S15.

$$\frac{dx^E}{dt} = -k_1 \cdot (x^E - x_{S1}^E) - k_2 \cdot (x^E - x_{S2}^E), \qquad (S12)$$

where the excess ^{13}C atom fractions of the two C sources are defined as

$$x_{S1}^E = x_{S1} - x_i \qquad (S13)$$

and

$$x_{S2}^E = x_{S2} - x_i. \qquad (S14)$$

Differential equation S12 describes how the isotopic composition of a cell varies over time if the cell assimilates C from two sources according to first-order kinetics.

Differential equation S12 is solved with the assumption that the isotopic composition of the C sources varies over time as described by equations S1 and S2. Without providing the mathematical details, we can assume that the solution takes the form

$$x^E(t) = A_0 + A_1 \cdot k \cdot t + A_2 \cdot e^{-k \cdot t},$$

where

$$k = k_1 + k_2 \qquad (S14)$$

is the rate constant of total C assimilation. By substituting this solution into equation S12 and applying the initial condition $x_i^E = 0$, we can solve for A_0, A_1, and A_2. These steps yield the following explicit formula for the function $x^E(t)$:

$$x^E(t) = \left(\frac{k_1}{k} \cdot x_{S1,i}^E + \frac{k_2}{k} \cdot x_{S2,i}^E\right) \cdot (1 - e^{-k \cdot t}) + \frac{k_1 \cdot K_{S1} + k_2 \cdot K_{S2}}{k^2} \cdot (k \cdot t - 1 + e^{-k \cdot t}), \qquad (S15)$$

Note that if C assimilation by the cell can be approximated by first-order kinetics, formula S15 is valid regardless of whether the cell divided during the time interval between 0 and t. Also note that expression S15 is equivalent to expression 15 in the main text if the isotopic composition of the two sources is time-independent (i.e., if K_{S1} and K_{S2} are equal to zero), as required.

1.1.3 Numerical examples

Here, the above results are illustrated with numerical examples. We consider two scenarios and plot the solutions separately for the zero-order and first-order kinetics of C assimilation.

In the first scenario, we assume that a cell assimilates C from only one source (i.e., $r_2 = 0$ and $k_2 = 0$), and the isotopic composition of the source increases or decreases linearly over time.[2] Specifically,

[2] This scenario can be a good approximation for SIP experiments where the isotopic composition of the substrate used for probing a specific activity (e.g., autotrophy, probed by the addition of ^{13}C-labeled inorganic C) varies due to the presence of other microorganisms and substrates in the sample. For example, heterotrophic microorganisms can respire organic C compounds naturally present in the sample, which will produce unlabeled inorganic C and thus decrease the

we assume that isotopic composition of the substrate changes at a rate of $K_{S1} = 0.003\ d^{-1}$ or $K_{S1} = -0.003\ d^{-1}$ from the initial value of $x_{S1,i} = 0.2$. For C assimilation modeled by zero-order kinetics, we assume the cell-specific assimilation rate of $r_1 = 1.35\ fmol\ C\ cell^{-1}\ h^{-1}$ and the average cellular C content of $\langle C \rangle = 10\ fmol\ C\ cell^{-1}$, which corresponds to a critical C content when the cell divides of $C_{max} \approx 13.9\ fmol\ C\ cell^{-1}$ and a doubling time of $\tau = C_{max}/(2 \cdot r_1) \approx 5.13\ h$. For C assimilation modeled by first-order kinetics, we assume the same value of $\langle C \rangle$ and the carbon-specific assimilation rate of $k_1 = 0.135\ h^{-1}$ (i.e., $k_1 = r_1/\langle C \rangle$). These values correspond to a critical C content of $C_{max} \approx 14.4\ fmol\ C\ cell^{-1}$ and the same doubling time of $\tau = \ln(2)/k_1 \approx 5.13\ h$. For both models we assume that the initial cellular C content is $C_i = C_{max}/2$, which corresponds to the initial cell cycle stage of $s_i = 0$, and calculate $x^E(t)$ over a time interval from 0 to $8 \cdot \tau$. This time interval was chosen so that the cell would divide 8 times to illustrate the differences in the shapes of the curves. Results of this simulation are shown in **Supplementary Figure S1**.

In the second scenario, we assume that a cell assimilates C from two labelled sources. The isotopic composition of the first source is assumed to linearly increase over time, whereas it is assumed to be constant for the second source.[3] Specifically, the isotopic composition of the first source is assumed to increase from the initial value of $x_{S1,i} = 0.011$ at a rate of $K_{S1} = 0.001\ h^{-1}$ or $K_{S1} = 0.004\ h^{-1}$, whereas the composition of the second source is assumed to be constant ($x_{S2,i} = 0.2$, $K_{S2} = 0$). For C assimilation modeled by zero-order kinetics, we assume the cell-specific assimilation rates of $r_1 = 1.35\ fmol\ C\ cell^{-1}\ h^{-1}$ and $r_2 = r_1/10 = 0.135\ fmol\ C\ cell^{-1}\ h^{-1}$ and the average cellular C content of $\langle C \rangle = 10\ fmol\ C\ cell^{-1}$, which corresponds to a critical C content when the cell divides of $C_{max} \approx 13.9\ fmol\ C\ cell^{-1}$ and a doubling time of $\tau = C_{max}/(2 \cdot (r_1 + r_2)) \approx 4.67\ h$. For C assimilation modeled by first-order kinetics, we assume the same value of $\langle C \rangle$ and the carbon-specific assimilation rates of $k_1 = r_1/\langle C \rangle = 0.135\ h^{-1}$ and $k_2 = r_2/\langle C \rangle = 0.0135\ h^{-1}$. These values correspond to the critical C content of $C_{max} \approx 14.4\ fmol\ C\ cell^{-1}$ and the same doubling time of $\tau = \ln(2)/(k_1 + k_2) \approx 4.67\ h$. In both cases we assume that the initial cellular C content is $C_i = C_{max}/2$, which corresponds to the initial cell cycle stage of $s_i = 0$, and calculate $x^E(t)$ over a time interval from 0 to $8 \cdot \tau$. This time interval was chosen so that the cell would divide 8 times to illustrate the differences in the curve shapes. Results of this simulation are shown in **Supplementary Figure S2**.

1.1.4 Calculation of rates

Equation S10 in this Supplement is similar to Eq. 11 in the main text. Thus, the approach for calculating r from $x^E_{S,i}$ based on Eq. S10 would be, in principle, similar to that described in the main text for zero-order kinetics of C assimilation. A notable difference would be that the zig-zag function Z would need

---

isotopic composition of the added ^{13}C-labeled inorganic C. Similarly, if ^{13}C-labeled organic C was added to the sample, it can be respired by heterotrophic microorganisms and thus increase the isotopic composition of the inorganic C pool.

[3] This scenario can be a good approximation for SIP experiments where, for instance, the target cells can assimilate two substrates (e.g., organic and inorganic C) and the target substrate (e.g., ^{13}C-labeled organic C) is continuously transformed into an alternative substrate (e.g., ^{13}C-labeled dissolved CO_2) by the activity of microorganisms that co-exist in the same sample as the target cells (see, e.g., Geerlings et al., 2020).

to include the factor between the large parentheses in Eq. S10. Implementation of this approach is not done here because it is beyond the scope of this study.

In contrast, Eq. S15 in this Supplement is much more complicated than Eq. 15 in the main text. Although Eq. S15 provides an explicit formula for calculating $x^E(t)$ from k, it is not possible to derive from it an explicit formula for calculating k from $x^E(t)$ (i.e., a formula similar to that shown in Eq. 20 in the main text). Thus, for first-order kinetics of C assimilation, estimation of k from $x^E(t)$ requires numerical iterations. Implementation of this approach is beyond the scope of this study.

1.2 Relationships between x_S^E, X_{net}, Fx_{net} and K_A

The *source-normalized excess* ^{13}C *atom fraction* defined in this study is inspired by the *excess* ^{13}C *atom fraction* (Coplen, 2011), which is commonly used in stable isotope research to quantify the difference between the ^{13}C atom fraction of a sample [here the cell sampled at the end of the SIP incubation, $x(t)$] and a standard (here the cell sampled prior to the SIP incubation, x_i):

$$x^E = x(t) - x_i.$$

However, for a cell assimilating C from a ^{13}C-labeled source, this difference will depend not only on the amount of C assimilated but also on the ^{13}C atom fraction of the carbon source, $x_{S,eff}$. To account for this dependence, we normalize the excess ^{13}C atom fraction of the *cell* by the excess ^{13}C atom fraction of the *source*, i.e.,

$$x_S^E(t) \equiv \frac{x(t)-x_i}{x_{S,eff}-x_i}.$$

This definition ensures that the final quantity, x_S^E, varies between 0 and 1 as the isotopic composition of the cell varies between x_i and $x_{S,eff}$. Note that, neglecting the effect of kinetic isotopic fractionation, the later value is the maximal ^{13}C atom fraction that the cell can reach by growing on a substrate characterized by $x_{S,eff}$.

To quantify the isotopic composition of a cell at the end of a SIP incubation, $x(t)$, we use an approach based on a specific C assimilation model (zero-order or first-order kinetics). This is because our aim is to link $x(t)$ with the *rate* of C assimilation. However, $x(t)$ can be derived *independently* of the C assimilation model, namely by performing mass balance calculations (e.g., Popa et al. 2007, Dekas et al. 2019). Denoting the initial C content and ^{13}C atom fraction of a cell as C_i and x_i, respectively, the initial amount of ^{13}C in the cell is $^{13}C_i = x_i \cdot C_i$. Similarly, denoting the amount of C assimilated by the cell during the SIP incubation as C_a and the ^{13}C atom fraction of the C source as $x_{S,eff}$, and neglecting kinetic isotopic fractionation, the amount of ^{13}C assimilated by the cell is $^{13}C_a = x_{S,eff} \cdot C_a$. Thus, if the cell did not divide during the incubation, its ^{13}C and total C content at the end of the SIP incubation are $^{13}C(t) = {}^{13}C_i + {}^{13}C_a$ and $C(t) = C_i + C_a$, respectively, which implies the following expression for the ^{13}C atom fraction:

$$\boldsymbol{x(t)} = \frac{\boldsymbol{^{13}C(t)}}{\boldsymbol{C(t)}} = \frac{\boldsymbol{C_i \cdot x_i + C_a \cdot x_{S,eff}}}{\boldsymbol{C_i + C_a}}.$$

By converting $x(t)$ from this equation to $x_S^E(t)$ defined above, we obtain the following expression:

$$x_S^E(t) = \frac{C_a}{C_i + C_a}.$$

This derivation shows that the source-normalized excess ^{13}C atom fraction defined in this study, x_S^E, is equivalent to the quantity X_{net} used by Dekas et al. (2019). Similarly, a simple rearrangement of the above formula shows that the quantity $x_S^E/(1 - x_S^E)$ is equivalent to the quantities Fx_{net} and K_A introduced by Popa et al. (2007) and Stryhanyuk et al. (2018), respectively. However, these relationships are only valid for a cell that did *not* divide during the SIP experiment. The approach developed in our study allows prediction of the isotopic composition of a cell over longer time scales, i.e., over multiple cell generations. This is especially important when the doubling time of the cell is comparable to, or shorter than, the duration of the SIP incubation but *a priori* unknown. This situation is likely when conducting a SIP experiment with a previously uncharacterized cell population (e.g., an environmental sample).

1.3 Estimating cellular C content

Quantification of the cell-specific C assimilation rate, *r*, for an individual cell requires a well constrained value of the cell's C content, including the C content of the very same cell for which the isotopic composition is measured by nanoSIMS, *C*, and the average C content of the cell from the same species as the cell measured by nanoSIMS, $\langle C \rangle$. Note that $\langle C \rangle$ refers to the cell's C content averaged over the *entire* cell cycle. Here we discuss conceptual approaches for estimating C and $\langle C \rangle$ that can readily be applied using cell cultures and planktic communities.

As previously done in several studies (Foster et al., 2013; Krupke et al., 2015; Schoffelen et al., 2018; Stryhanyuk et al., 2018; Mills et al., 2020; Trembath-Reichert et al., 2021), *C* may be estimated by multiplying the biovolume of the measured cell (*V*, in μm^3) with the corresponding carbon density (*ρ*, in mol C μm^{-3}). The biovolume can be estimated based on the shape and dimensions of the cell, which can be determined from the same nanoSIMS dataset as the ^{13}C atom fraction (e.g., from the $^{12}C_2^-$ or $^{12}C^{14}N^-$ ion count image). Ideally, carbon density should be obtained from a direct measurement, such as based on culture studies. Alternatively, carbon density may be estimated based on a similarity (e.g., phylogenetic and functional) of the measured cell to a species for which carbon density data is available in literature (e.g., Verity et al., 1992; Stryhanyuk et al., 2018; Khachikyan et al., 2019).

Determination of $\langle C \rangle$ requires availability of a culture of the same species as the cell measured by nanoSIMS. One way to determine $\langle C \rangle$ is to first ensure that the cell cycles in the cultured cell population are perfectly unsynchronized, and then measure, at an arbitrary time point, the bulk C content and cell counts. Note that such a single time point measurement will not yield the correct value for $\langle C \rangle$ if the cell cycles are partially synchronized. This is because the average C content per cell from such a population varies in time (**Supplementary Figure S4a-b**) and is therefore not representative of the average over the cell cycle.

If the cell cycles of the cultured cells are partially synchronized (as is often the case for photoautotrophs that tune cell division according to the circadian clock and to light-dark cycles), $\langle C \rangle$ can be estimated by measuring the C content of dividing cells, C_{max}. Our analysis of cells with partially synchronized cell cycles suggests that if cells in such a population divide, the cell size distribution (see Eq. **18** in the

main text) would appear as shown by the histograms in **Supplementary Figure S4a-b** (panels entitled C_0 = 6.9; note that the characteristic width of the distribution, ΔC, will depend on the degree of cell cycle synchronicity). Thus, by measuring the bulk C content, cell counts, and the cell size distribution around the time when most of the cells divide, one can estimate C_{max}. Based on this value, one can then calculate $\langle C \rangle$ according to $\langle C \rangle = C_{max}/\left(2 \cdot ln(2)\right) \approx 0.72 \cdot C_{max}$ (Eq. **17** in the main text) or $\langle C \rangle = C_{max} \cdot ln(2) \approx 0.69 \cdot C_{max}$ (Koch, 1966) depending on whether C assimilation by the cells can be approximated by zero-order or first-order kinetics, respectively.

1.4 Simulating SIP incubation

We used our model to simulate a SIP incubation and identify the most appropriate approach for estimating the average cell-specific rate of C assimilation in a population, $\langle r \rangle$, based on the *r* values determined for individual cells. We considered that the C contents and cell-specific C assimilation rates varied among cells in the population. For the C content, we assumed that the cells had either perfectly unsynchronized or partially synchronized cell cycles (see Eq. **16** and **18** in the main text, respectively). In both cases, we assumed the average C content of $\langle C \rangle$ = 10 fmol C cell^{-1}, which corresponds to the critical C content of C_{max} = 13.9 fmol C cell^{-1}. For the cell-specific rates, *r*, we assumed a log-normal distribution with the mean of 1.35 fmol C cell^{-1} h^{-1} and the standard deviation of 0.135 fmol C cell^{-1} h^{-1}. These values correspond to the average doubling time of $\tau \approx 5.1$ h. We assumed that binary cell division occurred when the cell's C content reached C_{max}, with C_{max} being equal among cells within the population. We additionally assumed that, during cell division, the *r* value of the mother cell is inherited by daughter cells. Finally, we assumed $x_{S,eff}$ = 0.2 and an initial cell abundance of N_i = 5000 cells (simulation results did not change for larger values of N_i; data not shown). At a specific time-point, *t*, we randomly sampled N=200 cells and tested different approaches to estimate $\langle r \rangle$. The approaches and the corresponding results are summarized in **Supplementary Figure S5.**

1.5 Modeling simultaneous assimilation of C and N

We use a simple mass balance model to simulate the effects of C and N assimilation by a cell from ^{13}C- and ^{15}N-labeled sources. First, the model quantifies the ^{13}C and ^{15}N atom fraction of a cell, denoted here as *x* and *y*, respectively, after certain amounts of C and N have been incorporated into the cell biomass or redistributed within the cell. The values of *x* and *y* are subsequently used to calculate the element-specific assimilation rates, k_C and k_N, using the approach described in the main text (Eq. **20**). The objective is to identify scenarios where the ratio of the calculated rates, k_C/k_N, deviates from 1.

As a case study we analyze cells of a unicellular diazotrophic cyanobacterium *Cyanothece* sp. ATCC 51142, which was recently studied in greater detail by Polerecky et al. (2021) and Rabouille et al. (2021). This species metabolizes the intracellular C and N storage pools, polysaccharide inclusions and cyanophycin granules, respectively, to manage requirements for C and N during growth. Thus, in our model the cell is divided into three compartments (**Supplementary Figure S6**): (i) polysaccharide inclusions, (ii) cyanophycin granules, and (iii) the cell matrix, which includes all other cell components.

Quantities in our model are given the subscripts *ps*, *cy*, and *m* when referring to polysaccharides, cyanophycin, and cell matrix, respectively. No subscript is given when referring to the whole cell. When referring to the initial value, quantities are additionally given the subscript *i*.

The initial amounts of C and N in each cell compartment are set to values listed in **Supplementary Table S1**. The initial isotopic composition of each compartment is set to the natural abundances:

$$x_i = x_{ps,i} = x_{cy,i} = x_{m,i} = 0.011$$

$$y_i = y_{cy,i} = y_{m,i} = 0.0037$$

To simulate C and N assimilation, C and N are taken from the external environment and added into each compartment. To simulate internal C and N recycling, C and N are transferred from one compartment to another. The added amounts are denoted as ΔC_d and ΔN_d, where the subscript *d* indicates the destination compartment. To ensure that the C:N of the destination compartment remains unchanged, the ratio of the added amounts of C and N must be equal to the C:N of the destination compartment:

polysaccharide inclusions:	$\Delta C_{ps} : \Delta N_{ps} = C_{ps,i} : N_{ps,ini} = 1{:}0 = \infty$,
cyanophycin granules:	$\Delta C_{cy} : \Delta N_{cy} = C_{cy,i} : N_{cy,i} = 2$,
cell matrix:	$\Delta C_m : \Delta N_m = C_{m,i} : N_{m,i} = 3.94$.

To simplify the model, the amounts of incorporated C and N are sufficiently small so that cell division can be ignored. Thus, the results are only applicable for time scales much shorter than the generation time. Additionally, we assume that the labeling of C and N transferred between internal C and N pools is negligible in comparison to the labeling of C and N added from the environment. Thus, in this simplified model, C and N are isotopically labeled when originating from the environment but are unlabeled when originating from an internal C and N pool.

Under these assumptions, the final amounts of ^{13}C, total C (i.e., ^{12}C + ^{13}C), ^{15}N and total N (i.e., ^{14}N + ^{15}N) in each compartment and in the whole cell are calculated as follows:

polysaccharide inclusions:	$C_{ps} = C_{ps,i} + \Delta C_{ps}$	$^{13}C_{ps} = {}^{13}C_{ps,i} + x_{S,eff(ps)} \cdot \Delta C_{ps}$
cyanophycin granules:	$C_{cy} = C_{cy,i} + \Delta C_{cy}$	$^{13}C_{cy} = {}^{13}C_{cy,i} + x_{S,eff(cy)} \cdot \Delta C_{cy}$
	$N_{cy} = N_{cy,i} + \Delta N_{cy}$	$^{15}N_{cy} = {}^{15}N_{cy,i} + y_{S,eff(cy)} \cdot \Delta N_{cy}$
cell matrix:	$C_m = C_{m,i} + \Delta C_m$	$^{13}C_m = {}^{13}C_{m,i} + x_{S,eff(m)} \cdot \Delta C_m$
	$N_m = N_{m,i} + \Delta N_m$	$^{15}N_m = {}^{15}N_{m,i} + y_{S,eff(m)} \cdot \Delta N_m$
whole cell:	$C = C_{ps} + C_{cy} + C_m$	$^{13}C = {}^{13}C_{ps} + {}^{13}C_{cy} + {}^{13}C_m$
	$N = N_{ps} + N_{cy} + N_m$	$^{15}N = {}^{15}N_{ps} + {}^{15}N_{cy} + {}^{15}N_m$

In these equations, $x_{S,eff(d)}$ and $y_{S,eff(d)}$ denote the isotopic composition of the *effective* source from which C and N are added to the destination compartment *d*. Their values differ depending on the simulated scenario (see below).

Finally, the ^{13}C and ^{15}N atom fractions of the whole cell are calculated from the corresponding amounts of ^{13}C, total C, ^{15}N and total N based on the definition: $x = {}^{13}C/C$ and $y = {}^{15}N/N$. These values are then used to calculate the element-specific assimilation rates, k_C and k_N, as described in the main text (Steps 1 and 2, Eq. **10** and **20**) assuming incubation time of 1 h.

1.5.1 Simulation 1

This simulation involves C and N assimilation by a cell without storage inclusions (**Supplementary Figure S6a**). Assimilated C and N originate from two different sources: isotopically labeled target sources ($x_{S,tar} = 1$, $y_{S,tar} = 1$) and unlabeled alternative sources ($x_{S,alt} = 0.011$, $y_{S,alt} = 0.0037$). The amount of added C and N is $\Delta C_m = 14.1$ fmol and $\Delta N_m = 3.58$ fmol, respectively, which corresponds to a 10% increase in the cell matrix biomass. The contribution of the target source to the total incorporated C is $f_{C,tar} = 0.8$ or 1 and is similar for N ($f_{N,tar} = 0.8$ or 1). However, calculation of the element-specific rates k_C and k_N assumes that both C and N are only assimilated from the target source ($x_{S,eff} = 1$, $y_{S,eff} = 1$). Results show that k_C/k_N deviate from 1 if incorporation of C and N into the cell involves more than one source (main text, **Figure 8, stars**). Specifically, $k_C/k_N > 1$ if cellular assimilation of N involved more than one source, while $k_C/k_N < 1$ if cellular assimilation of C involved more than one source.

1.5.2 Simulation 2

This simulation involves C incorporation into polysaccharide inclusions (**Supplementary Figure S6b**). Assimilated C originates from one source, which is isotopically labeled ($x_{S,eff} = 1$), and the amount of added C is $\Delta C_{ps} = 12.5$ fmol, which corresponds to a 10% increase in the polysaccharide content in the cell. The result of this simulation shows that k_C/k_N approaches infinity because the cell only incorporated C and no N (main text, **Figure 8, triangle**).

1.5.3 Simulation 3

This simulation involves C and N incorporation into cyanophycin granules (**Supplementary Figure S6b**). Assimilated C originates from one source, which is isotopically labeled ($x_{S,eff} = 1$) and is similar for N ($y_{S,eff} = 1$). The amount of added C and N is $\Delta C_{cy} = 6.6$ fmol and $\Delta N_{cy} = 3.3$ fmol, respectively, which corresponds to a 5-fold increase in the cyanophycin content in the cell. The result shows that $k_C/k_N < 1$ (main text, **Figure 8, circle**). This result is caused by C and N being incorporated into a compartment with a lower C:N ratio than the whole cell ($\Delta C_{cy}/\Delta N_{cy} < C_i/N_i$). In other words, the cell assimilated relatively more N than C than would be predicted by its initial C:N content.

1.5.4 Simulation 4

This simulation involves C and N incorporation into the matrix of a cell *with* storage inclusions (**Supplementary Figure S6b**). Assimilated C originates from one source, which is isotopically labeled ($x_{S,eff} = 1$) and is similar for N ($y_{S,eff} = 1$). The amount of added C and N is $\Delta C_m = 14.1$ fmol and $\Delta N_m = 3.58$ fmol, respectively, which corresponds to a 10% increase in the cell matrix biomass. The result shows that $k_C/k_N < 1$ (main text, **Figure 8, square**). This result is caused by C and N being incorporated into a compartment with a lower C:N ratio than the whole cell ($\Delta C_m/\Delta N_m < C_i/N_i$), i.e., the cell assimilated relatively more N than C than would be predicted by its initial C:N content. Furthermore,

k_C/k_N in simulation 4 is closer to 1 than k_C/k_N in simulation 3 because the C:N ratio in the cell matrix is closer to that of the whole cell ($C_m/N_m > C_{cy}/N_{cy}$).

1.5.5 Simulation 5

This simulation involves C and N incorporation into cyanophycin granules using internally recycled C and N in addition to C and N originating from the target sources provided externally. Unlabeled internal C and N sources are derived from polysaccharides and catabolized proteins in the cell matrix, respectively (**Supplementary Figure S6c**). The target external C and N sources are isotopically labeled ($x_{S,tar}$ = 1, $y_{S,tar}$ = 1), whereas the internally recycled C and N are unlabeled ($x_{S,alt}$ = 0.011, $y_{S,alt}$ = 0.0037). The amount of added C and N is ΔC_{cy} = 6.6 fmol and ΔN_{cy} = 3.3 fmol, respectively, which corresponds to a 5-fold increase in the cyanophycin content in the cell. The relative contribution of the external C source is fixed ($f_{C,tar}$ = 0.5), whereas the contribution of the external N source is $f_{N,tar}$ = 0.05, 0.5, 0.8 or 1. However, calculation of the element-specific rates k_C and k_N assumes that both C and N are only assimilated from the target source ($x_{S,eff}$ = 1, $y_{S,eff}$ = 1). Results of this simulation show that $k_C/k_N < 1$ for most values of $f_{N,tar}$ (main text, **Figure 8, diamonds**). This result is caused by C and N being incorporated into a compartment with a lower C:N ratio than the whole cell (compare with **Simulation 3**). However, k_C/k_N may increase above 1 if $f_{N,tar}$ is sufficiently low and $f_{C,tar}$ is sufficiently high (main text, **Figure 8, left-most diamond**).

2 Supplementary Tables and Figures

2.1 Supplementary Table S1.

C and N content in cells of *Cyanothece* sp. ATCC 51142. Values are adopted from Rabouille et al. (2021) and correspond to the average cell harvested from the diazotrophic culture at the end of the 14 h light period.

**Cell compartment**	**C content** (fmol C $cell^{-1}$)	**N content** (fmol N $cell^{-1}$)
polysaccharide inclusions	125	-
cyanophycin granules	1.32	0.66
cell matrix	141	35.8
whole cell	268	36.4

2.2 Supplementary Figure S1.

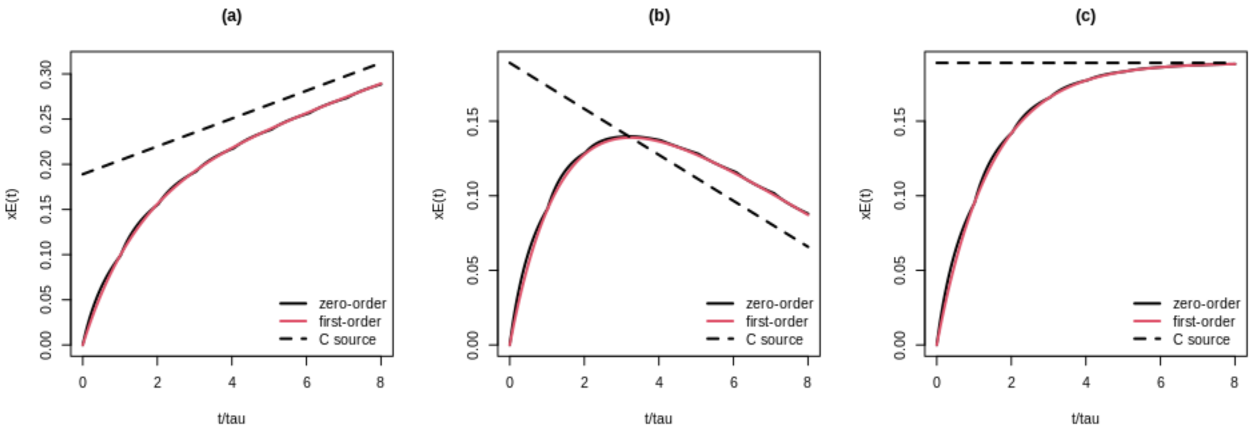

Modeled isotopic composition of a cell assimilating a substrate with a time-dependent isotopic composition. The isotopic composition is expressed as excess ^{13}C atom fraction, x^E. Solid and dashed lines correspond to the cell and substrate, respectively. The isotopic composition of the substrate linearly increases (panel **a**) or decreases (panel **b**) over time. For comparison, the situation corresponding to a substrate with a constant isotopic composition is shown in panel **c**. Black and red solid lines correspond to zero-order (Eq. **S9**) and first-order (Eq. **S15**) kinetics of C assimilation, respectively.

2.3 Supplementary Figure S2.

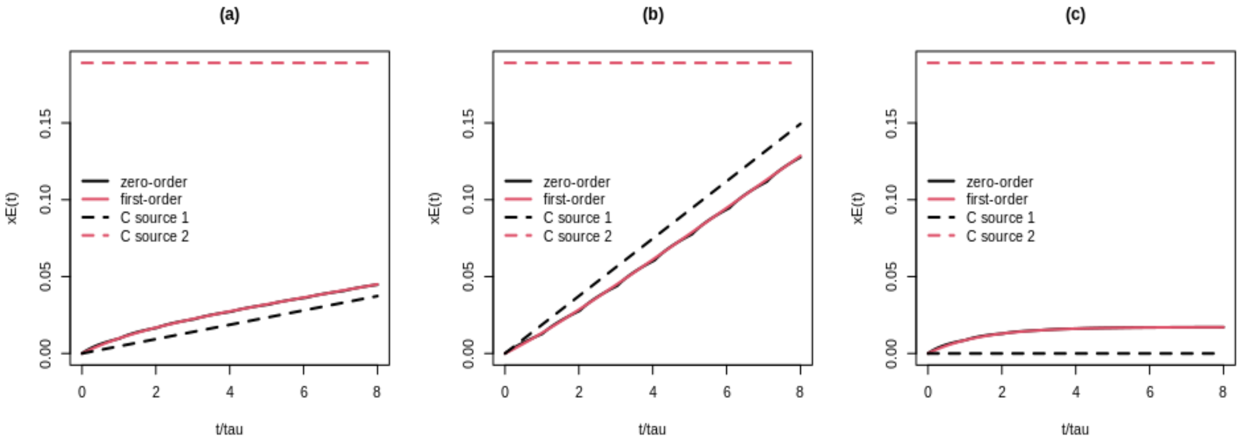

Modeled isotopic composition of a cell assimilating C from two sources. The isotopic composition is expressed as excess ^{13}C atom fraction, x^E. Solid and dashed lines correspond to the cell and substrate, respectively. The isotopic composition of the first C source (black) increases over time at different rates ($K_{S1} = 0.001\ h^{-1}$ in panel **a**, $K_{S1} = 0.004\ h^{-1}$ in panel **b**), while the composition of the second source (red) is constant. For comparison, the situation where both sources have constant isotopic composition is shown in panel **c**. Black and red lines correspond to the zero-order (Eq. **S9**) and first-order (Eq. **S15**) kinetics of C assimilation, respectively.

2.4 Supplementary Figure S3.

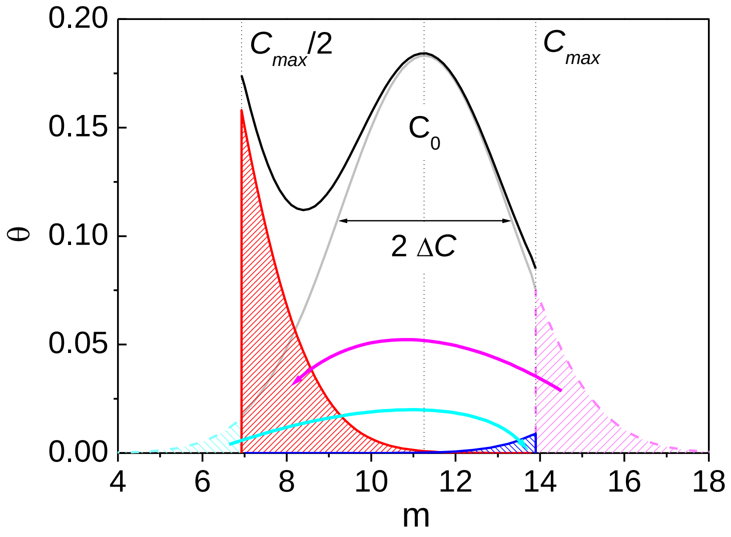

Schematic diagram showing the process of deriving the probability density function (PDF) for partially synchronized cells. The PDF, denoted as θ, describes the distribution of cellular C content in a population of cells with partially synchronized cell cycles, where each cell assimilates C with the same cell-specific rate, r (zero-order kinetics). Shown is the PDF at a given point in time. The PDF is based on the Gaussian function $exp[-(m - C_0)^2/(2 \cdot \Delta C^2)]$, where C_0 is the central value and ΔC is the width of the function. However, because θ is non-zero only within the interval between $C_{max}/2$ and C_{max}, the tails of the Gaussian function reaching outside this interval must be modified. Specifically, the right tail ($C_{max} < C < 1.5 \cdot C_{max}$; **magenta**) must be shifted to the left and multiplied by a factor of 2 (**red**) to account for binary division. Similarly, the left tail ($0 < C < C_{max}/2$; **cyan**) must be shifted to the right and multiplied by a factor of 1/2 (**blue**) to account for binary division. Similar treatment is applied to tails reaching above $C_{max} + \mathrm{j} \cdot C_{max}/2$ and below $C_{max}/2 - j \cdot C_{max}/2$, where $j = 2, 3, \ldots$, except the factor becomes 2^j and $1/2^j$, respectively (not shown in the diagram). No modification is needed for the central part of the Gaussian function within the interval between $C_{max}/2$ and C_{max} (**gray**). The final θ is given by the sum of the central part and the shifted tails (**black**; Eq. **18** in the main text). Population growth according to zero-order kinetics is modeled by considering that ΔC remains constant while the central value C_0 increases linearly in time, $C_0(t) = \mathrm{C}_{0,\mathrm{i}} + r \cdot t$, and is reset to $C_{max}/2$ when reaching C_{max}, where $C_{0,i}$ is the initial value and r is the cell-specific growth rate (assumed to be equal for all cells in the population). If ΔC is small compared to $C_{max}/2$, 95% of cells in the population divide during the time interval between $(C_0 + 2 \cdot \Delta C)/r$ and $(C_0 - 2 \cdot \Delta C)/r$. This is because 95% of the area under the Gaussian curve lies in the interval between $C_0 - 2 \cdot \Delta C$ and $C_0 + 2 \cdot \Delta C$. Thus, the ratio between the population doubling time, $\tau = C_{max}/(2 \cdot r)$, and the time interval during which 95% of cells divide, $\Delta t_{95} = 4\Delta C/r$, is a measure of *cell cycle synchronicity* in the population: $\gamma = \tau/\Delta t_{95} = C_{max}/(8 \cdot \Delta C)$.

2.5 Supplementary Figure S4.

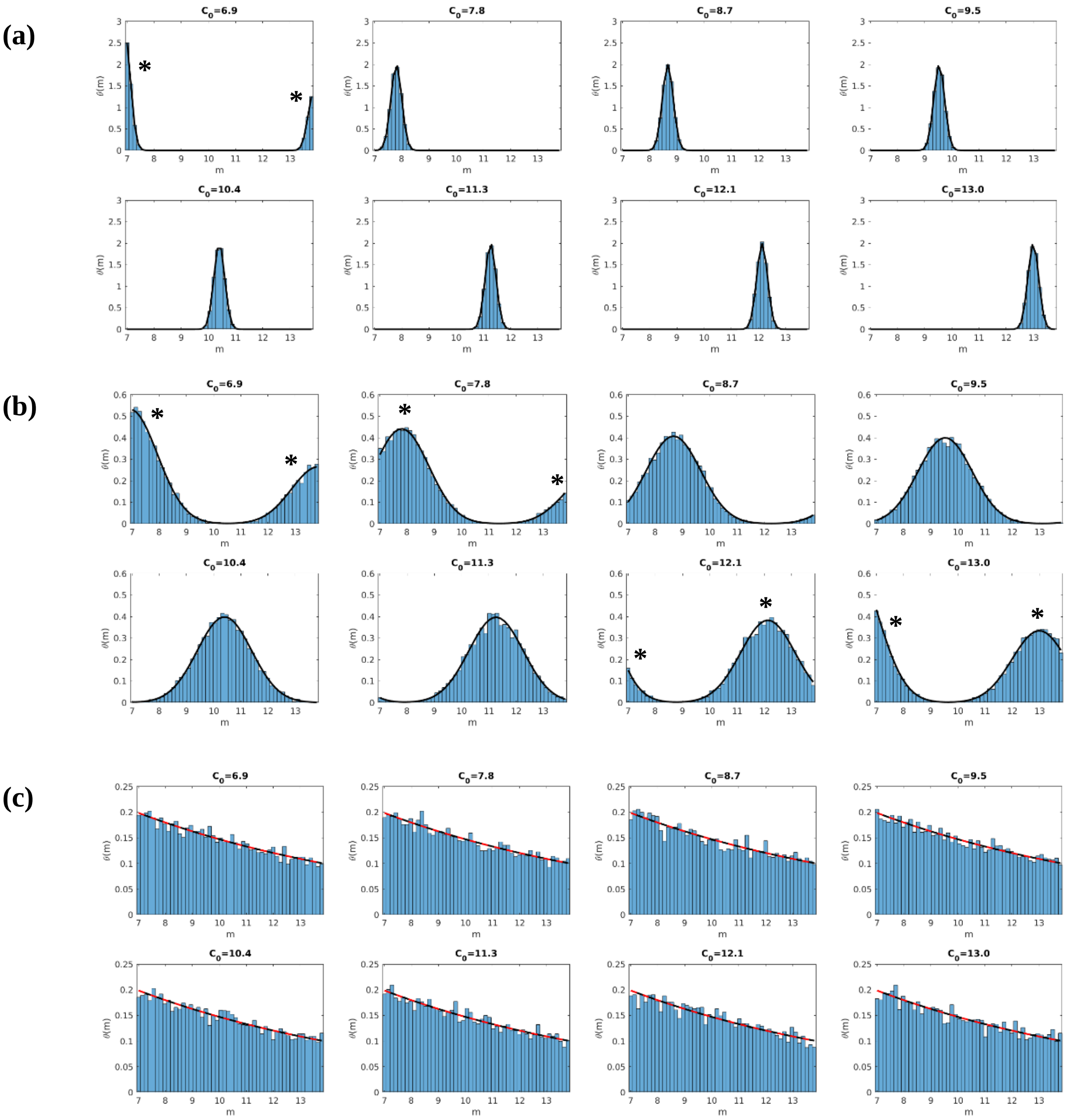

Examples of probability density functions (PDFs) at different time points and for cell populations with different degrees of cell cycle synchronicity. The PDFs describe the distribution of cellular C content (x-axis) in a population of cells with partially synchronized cell cycles. Each cell in the population assimilates C with the same cell-specific rate, *r* (zero-order kinetics). Shown are the PDFs (**black lines**) and the corresponding histograms (**blue bars**) for cells randomly selected from the population. Calculated for $\langle C \rangle$ = 10 fmol C cell^{-1}, for which the cellular C content varies between $C_{max}/2$ = 6.93 fmol C and C_{max} = 13.86 fmol C (Eq. **17** in the main text). Different panels show populations characterized with a different degree of cell cycle synchronicity: (**a**) very synchronized cell, γ = 8.7; (**b**) moderately synchronized cells, γ = 1.7; and (**c**) poorly synchronized cells, γ = 0.35. These degrees of synchronicity correspond to the following widths of the Gaussian function in Eq. **18** in the main text

(see also **Supplementary Figure S3**): $\Delta C = 0.2$, $\Delta C = 1$ and $\Delta C = 5$ fmol C cell^{-1}, respectively. In all cases, cells grow at the same cell-specific rate $r = 6.93$ fmol C cell^{-1} d^{-1}, which corresponds to the population doubling time $\tau = 1$ d. Growth of the population is modeled by considering that C_0 increases linearly in time, $C_0(t) = C_{max}/2 + r \cdot t$, when $C(t) < C_{max}$, and C_0 is reset to $C_{max}/2$ when it reaches C_{max}. Thus, the different graphs show the PDFs at time points $t_j = j \cdot \tau/8$, where $j = 0, 1, \ldots, 7$. The values of C_0 (in fmol C) corresponding to these time points are given in the title of each graph. Note that cells belong to *one* population (characterized by r, $\langle C \rangle$ and γ) although the histograms of C at certain time points (marked by **stars**) appear to show two sub-populations, one "localized" close to $C_{max}/2$ and another one "localized" close to C_{max}. This time-dependent "splitting" of one population into two apparent sub-populations is a consequence of cell division. Also note that for the lowest γ (i.e., the highest value of ΔC; panel **c**), the distribution resembles that for perfectly unsynchronized cells (**red dashed line**; Eq. **16** in the main text). In this case the PDF is time independent.

2.6 Supplementary Figure S5.

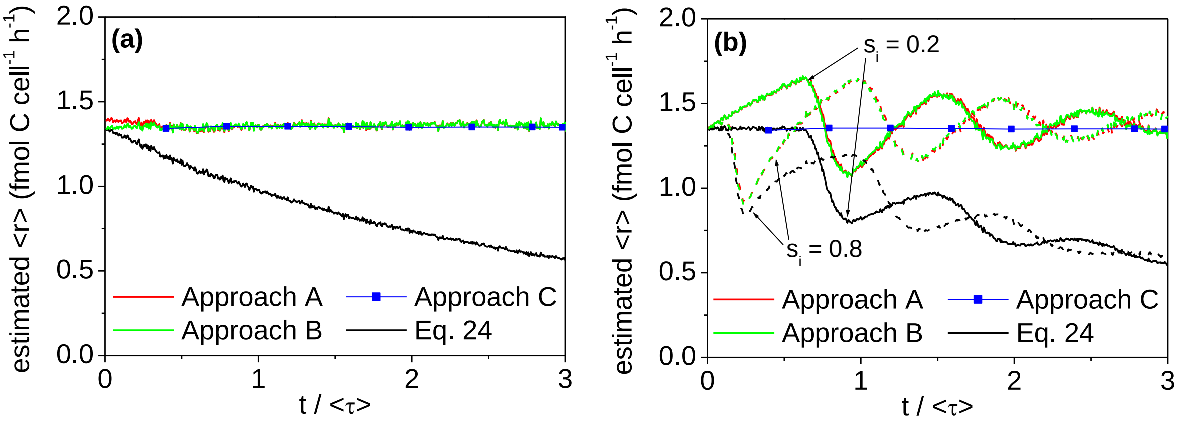

Comparison of different approaches for estimating the average cell-specific C assimilation rate in a cell population, $\langle r \rangle$. Results are shown separately for cells with perfectly unsynchronized (panel **a**) and partially synchronized (panel **b**) cell cycles, and the estimated $\langle r \rangle$ values are shown as a function of incubation time expressed relative to the average population doubling time ($t/\langle \tau \rangle$). In the simulation, the true mean of the population was $\langle r \rangle = 1.35$ fmol C cell^{-1} h^{-1}. In the approaches depicted by **red**, **green**, and **blue** lines (note that the green and red lines almost perfectly overlap), r values in individual cells were calculated according to Approach A, B and C (see main text), respectively, and averaged over sampled cells. For a population with perfectly unsynchronized cell cycles, the latter approaches correctly estimate the true population average across a broad range of incubation times (panel **a**). For a population with partially synchronized cell cycles, Approaches A and B yield similar estimates of $\langle r \rangle$, but these estimates oscillate by roughly ± 25% around the true population mean depending on the incubation time and the initial cell cycle stage of cells in the population (**red** and **green** lines in panel **b**). In contrast, the average of r values calculated by Approach C correctly estimates the true population mean across a broad range of incubation times (**blue** line in panel **b**). In the approach depicted by **black** lines, $\langle r \rangle$ was estimated by averaging r values in individual cells calculated according to Eq. **24** (see

main text). This approach underestimates the true population average if cells divided during the incubation. For a population with perfectly unsynchronized cell cycles, underestimation occurs when the incubation time exceeds about 10% of the average doubling time ($t/\langle\tau\rangle > 0.1$; black line in panel **a**). For a population with partially synchronized cell cycles, underestimation occurs if the incubation time roughly exceeds $\langle\tau\rangle \cdot (1 - s_i)$, where s_i is the average cell cycle stage of cells in the population at the beginning of incubation (compare solid and dashed black lines in panel **b**). Additionally, the deviation between the true and estimated $\langle r\rangle$ increases with the incubation time.

2.7 Supplementary Figure S6.

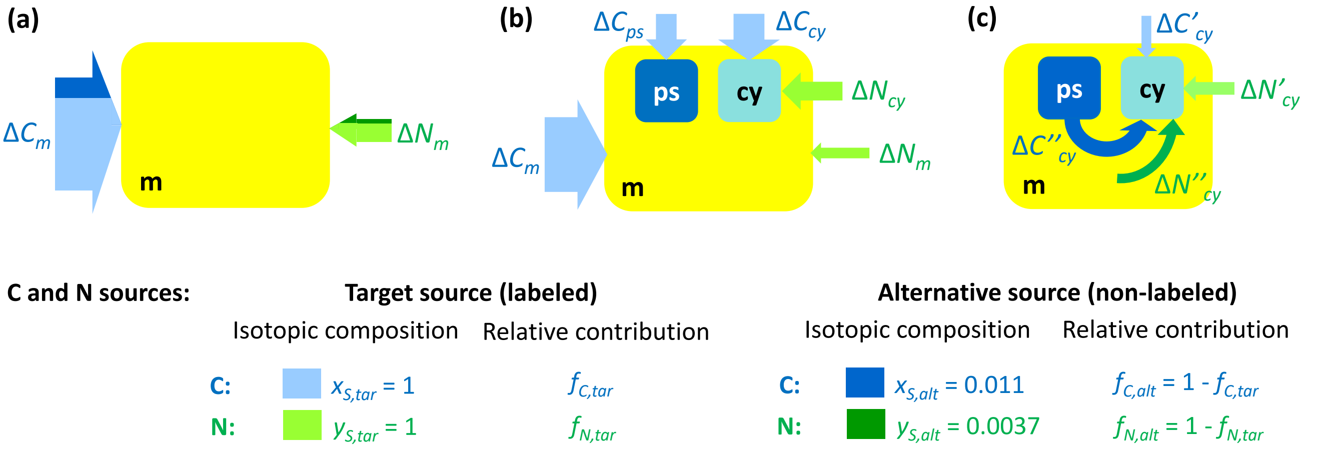

Conceptual diagrams used in simulations of C and N assimilation by a cell. The cell is divided into three compartments: the pool of C storage compounds (e.g., polysaccharides, **ps**), the pool of N storage compounds (e.g., cyanophycin, **cy**), and the cell matrix (**m**) containing all other cell components. Arrows indicate inputs of C and N. The lighter and darker colors correspond to the isotopically labeled target sources and the unlabeled alternative sources, respectively. Panels illustrate C and N flow in different simulated scenarios: **(a)** C and N assimilation by a cell without storage inclusions (**Simulation 1**); **(b)** C incorporation into polysaccharide inclusions (**Simulation 2**), C and N incorporation into cyanophycin granules (**Simulation 3**) and into the matrix of a cell with storage inclusions (**Simulation 4**); **(c)** C and N incorporation into cyanophycin granules using internally recycled C and N in addition to C and N originating from the target sources provided externally (**Simulation 5**).

3 Supplementary References

Coplen, T. B. (2011). Guidelines and recommended terms for expression of stable-isotope-ratio and gas-ratio measurement results. *Rapid Commun. Mass Spectrom.* 25, 2538–2560. doi:10.1002/rcm.5129.

Dekas, A. E., Parada, A. E., Mayali, X., Fuhrman, J. A., Wollard, J., Weber, P. K., et al. (2019). Characterizing Chemoautotrophy and Heterotrophy in Marine Archaea and Bacteria With Single-Cell Multi-isotope NanoSIP. *Front. Microbiol.* 10, 2682. Available at: https://www.frontiersin.org/article/10.3389/fmicb.2019.02682.

Foster, R. A., Sztejrenszus, S., and Kuypers, M. M. M. (2013). Measuring carbon and N2 fixation in field populations of colonial and free-living unicellular cyanobacteria using nanometer-scale secondary ion mass spectrometry. *J. Phycol.* 49, 502–516. doi:10.1111/jpy.12057.

Geerlings, N. M. J., Karman, C., Trashin, S., As, K. S., Kienhuis, M. V. M., Hidalgo-Martinez, S., et al. (2020). Division of labor and growth during electrical cooperation in multicellular cable bacteria. *Proc. Natl. Acad. Sci.* 117, 5478. doi:10.1073/pnas.1916244117.

Khachikyan, A., Milucka, J., Littmann, S., Ahmerkamp, S., Meador, T., Könneke, M., et al. (2019). Direct Cell Mass Measurements Expand the Role of Small Microorganisms in Nature. *Appl. Environ. Microbiol.* 85, 1–1. doi:10.1128/AEM.00493-19.

Koch, A. L. (1966). Distribution of Cell Size in Growing Cultures of Bacteria and the Applicability of the Collins-Richmond Principle. *J. Gen. Microbiol.* 45, 409–417. doi:10.1099/00221287-45-3-409.

Krupke, A., Mohr, W., LaRoche, J., Fuchs, B. M., Amann, R. I., and Kuypers, M. M. M. (2015). The effect of nutrients on carbon and nitrogen fixation by the UCYN-A–haptophyte symbiosis. *ISME J.* 9, 1635–1647. doi:10.1038/ismej.2014.253.

Mills, M. M., Turk-Kubo, K. A., van Dijken, G. L., Henke, B. A., Harding, K., Wilson, S. T., et al. (2020). Unusual marine cyanobacteria/haptophyte symbiosis relies on N2 fixation even in N-rich environments. *ISME J.* doi:10.1038/s41396-020-0691-6.

Polerecky, L., Masuda, T., Eichner, M., Rabouille, S., Vancová, M., Kienhuis, M. V. M., et al. (2021). Temporal Patterns and Intra- and Inter-Cellular Variability in Carbon and Nitrogen Assimilation by the Unicellular Cyanobacterium Cyanothece sp. ATCC 51142. *Front. Microbiol.* 12. doi:10.3389/fmicb.2021.620915.

Popa, R., Weber, P. K., Pett-Ridge, J., Finzi, J. A., Fallon, S. J., Hutcheon, I. D., et al. (2007). Carbon and nitrogen fixation and metabolite exchange in and between individual cells of Anabaena oscillarioides. *ISME J.* 1, 354–360. doi:10.1038/ismej.2007.44.

Rabouille, S., Campbell, D. A., Masuda, T., Zavřel, T., Bernát, G., Polerecky, L., et al. (2021). Electron & Biomass Dynamics of Cyanothece Under Interacting Nitrogen & Carbon Limitations. *Front. Microbiol.* 12. doi:10.3389/fmicb.2021.617802.

Schoffelen, N. J., Mohr, W., Ferdelman, T. G., Littmann, S., Duerschlag, J., Zubkov, M. V., et al. (2018). Single-cell imaging of phosphorus uptake shows that key harmful algae rely on different phosphorus sources for growth. *Sci. Rep.* 8. doi:10.1038/s41598-018-35310-w.

Stryhanyuk, H., Calabrese, F., Kümmel, S., Musat, F., Richnow, H. H., and Musat, N. (2018). Calculation of Single Cell Assimilation Rates From SIP-NanoSIMS-Derived Isotope Ratios: A Comprehensive Approach. *Front. Microbiol.* 9, 2342. Available at: https://www.frontiersin.org/article/10.3389/fmicb.2018.02342.

Trembath-Reichert, E., Shah Walter, S. R., Ortiz, M. A. F., Carter, P. D., Girguis, P. R., and Huber, J. A. (2021). Multiple carbon incorporation strategies support microbial survival in cold subseafloor crustal fluids. *Sci. Adv.* 7, eabg0153. doi:10.1126/sciadv.abg0153.

Verity, P. G., Robertson, C. Y., Tronzo, C. R., Andrews, M. G., Nelson, J. R., and Sieracki, M. E. (1992). Relationships between cell volume and the carbon and nitrogen content of marine photosynthetic nanoplankton. *Limnol. Oceanogr.* 37, 1434–1446. doi:10.4319/lo.1992.37.7.1434.
